# Natural and Anthropogenic Hybridization in Two Species of Eastern Brazilian Marmosets (*Callithrix jacchus* and *C*. *penicillata*)

**DOI:** 10.1371/journal.pone.0127268

**Published:** 2015-06-10

**Authors:** Joanna Malukiewicz, Vanner Boere, Lisieux F. Fuzessy, Adriana D. Grativol, Ita de Oliveira e Silva, Luiz C. M. Pereira, Carlos R. Ruiz-Miranda, Yuri M. Valença, Anne C. Stone

**Affiliations:** 1 Departamento de Bioquímica e Biologia Molecular, Universidade Federal de Viçosa, Viçosa MG, Brazil; 2 School of Human Evolution and Social Change, Arizona State University, Tempe, Arizona, 85287, United States of America; 3 Department of Plant Biology, Universidade Federal de Minas Gerais, Belo Horizonte MG, Brazil; 4 Ciências Ambientias, Centro de Biociências e Biotecnologia, Universidade Estadual do Norte Fluminense, Campos dos Goytacazes RJ, Brazil; 5 Departamento de Biologia Animal, Universidade Federal de Viçosa, Viçosa MG, Brazil; 6 Centro de Conservação e Manejo de Fauna da Caatinga, Universidade Federal do Vale do São Francisco, Petrolina PE, Brazil; 7 Centro de Reabilitação de Animais Silvestres do Santuário dos Três Reinos, Recife PE, Brazil; Queensland University of Technology, AUSTRALIA

## Abstract

Animal hybridization is well documented, but evolutionary outcomes and conservation priorities often differ for natural and anthropogenic hybrids. Among primates, an order with many endangered species, the two contexts can be hard to disentangle from one another, which carries important conservation implications. *Callithrix* marmosets give us a unique glimpse of genetic hybridization effects under distinct natural and human-induced contexts. Here, we use a 44 autosomal microsatellite marker panel to examine genome-wide admixture levels and introgression at a natural *C*. *jacchus* and *C*. *penicillata* species border along the São Francisco River in NE Brazil and in an area of Rio de Janeiro state where humans introduced these species exotically. Additionally, we describe for the first time autosomal genetic diversity in wild *C*. *penicillata* and expand previous *C*. *jacchus* genetic data. We characterize admixture within the natural zone as bimodal where hybrid ancestry is biased toward one parental species or the other. We also show evidence that São Francisco River islands are gateways for bidirectional gene flow across the species border. In the anthropogenic zone, marmosets essentially form a hybrid swarm with intermediate levels of admixture, likely from the absence of strong physical barriers to interspecific breeding. Our data show that while hybridization can occur naturally, the presence of physical, even if leaky, barriers to hybridization is important for maintaining species genetic integrity. Thus, we suggest further study of hybridization under different contexts to set well informed conservation guidelines for hybrid populations that often fit somewhere between “natural” and “man-made.”

## Introduction

While animal hybridization is historically regarded as an “evolutionary dead-end” [1], contemporary research takes a more multi-faceted view, particularly in terms of natural versus anthropogenic hybridization. Here, we define hybridization as successful interbreeding between individuals from different populations (usually subspecies or species) possessing distinguishable heritable characteristics (modified from [1]). We also differentiate between natural and anthropogenic hybridization, with the latter as population interbreeding resulting from human-induced environmental change. Natural hybrid zones are often heralded as “nature’s evolutionary laboratories,” through growing evidence for the importance of animal hybridization in speciation, introgression (gene transfer between taxa), and development of genetic novelties (e.g. [2, 3, 4]). Natural hybridization may also be a regular part of species divergence in young taxa and occurs in approximately 10% of animal species [3]. On the other hand, biodiversity declines and erosion of species genetic integrity are often linked to anthropogenic hybridization [5, 6]. Instances of hybrid swarming where highly admixed populations lose unique parental gene combinations are also attributed to anthropogenic hybridization (e.g. [7, 8]). Surprisingly, some cases of anthropogenic hybridization may increase biodiversity through hybrid speciation and transgressive segregation [9].

Allendorf *et al*. [10] perhaps best summarize current views regarding hybridization, where the authors acknowledge its importance in animal evolutionary history within a natural context, but emphasize its negative impact on modern biodiversity due to anthropogenic factors. This dichotomous thought regarding hybridization makes conservation decisions especially difficult, particularly when distinguishing between the two processes is not simple. Accordingly, Allendorf *et al*. [10] highlight the importance of understanding the evolutionary role of hybridization in light of management of interbreeding taxa. More specifically, the authors recommend conservation of all naturally admixed populations. In contrast, conservation of anthropogenically admixed populations should be considered only when few, if any, pure populations remain.

The challenges described by Allendorf *et al*. [10] certainly pertain to primates, an order with many documented cases of hybridization (e.g. [11, 12]). However, the anthropogenic and natural components driving primate hybridization can be hard to disentangle from one another, as widely illustrated by African cercopithecines [11, 13]. While hybridization is inherently neither good nor bad, understanding the respective evolutionary consequences of human and natural factors has important conservation implications for primates, particularly as 32.8% of primate species are listed as endangered [14]. Thus, it would be ideal to examine and compare the genetic and evolutionary effects of primate hybridization between clearly defined anthropogenic and natural contexts.

Hybridization among eastern Brazilian marmosets (genus *Callithrix*) provides a unique opportunity to study interspecific breeding within separate natural and anthropogenic contexts. *Callithrix* contains six species, *C*. *jacchus*, *C*. *penicillata*, *C*. *geoffroyi*, *C*. *kuhlii*, *C*. *flaviceps*, and *C*. *aurita*, with the last three species being threatened or near threatened (www.iucnredlist.org). This is a young genus, aged at about 2.5 million years, with *C*. *penicillata* and *C*. *jacchus* diverging as sister species less than 1 million years ago [15]. Experimental hybridization in captivity shows incomplete reproductive isolation between various members of *Callithrix* [16]. All species inhabit separate ranges within the Caatinga, Cerrado, and Brazilian Atlantic Forest biomes of Brazil, but inter-specific points of contact do occur at species boundaries [17]. *Callithrix jacchus* and *C*. *penicillata* have also been introduced together in areas outside of their respective distributions, and into the native ranges of other *Callithrix* species. Marmoset hybridization occurs at many of these natural and anthropogenic contact points (e.g. [18, 19, 20, 21, 22]).

We have previously characterized genetic diversity and introgression at a putative natural and anthropogenic *C*. *penicillata* x *C*. *jacchus* hybrid zone in NE and SE Brazil, respectively, using the mitochondrial DNA control region (mtDNA CR) [23]. Here, we reexamined hybridization within the same *C*. *penicillata* x *C*. *jacchus* natural and anthropogenic hybrid zones using a large genome-wide panel of autosomal microsatellites. These autosomal markers complemented the mtDNA data [23], which were based on a single-locus that tracks female-only gene flow in primates, to reveal a more comprehensive view of hybridization throughout the nuclear genome and gene flow for both sexes. We used the expanded genetic marker set in hybrid and reference parental populations to address the following questions: (1) What are autosomal genetic diversity and differentiation patterns inside and outside of *C*. *jacchus x C*. *penicillata* hybrid zones? (2) Do levels of genetic admixture and introgression differ between natural and anthropogenic marmoset hybrid zones? (3) What are the evolutionary and conservation implications of natural and anthropogenic marmoset hybridization?

Our large microsatellite dataset gave first time estimates of autosomal genetic diversity for any wild *C*. *penicillata* populations and expanded previous *C*. *jacchus* genetic data. We characterized individual admixture within the natural hybrid zone as biased toward one parental species or the other, likely the result of a large river system flowing through this zone and limiting interspecific gene flow. Interestingly, fluvial islands within this river system may serve as gateways for bidirectional gene flow across the species border. On the other hand, marmosets essentially formed a hybrid swarm in the anthropogenic zone and showed intermediate levels of admixture. The anthropogenic hybrid zone admixture and swarm patterns have probably resulted from the absence of a strong physical barrier to limit interbreeding between introduced *C*. *jacchus* and *C*. *penicillata*.

## Materials and Methods

### Sample populations

**Fig 1:**
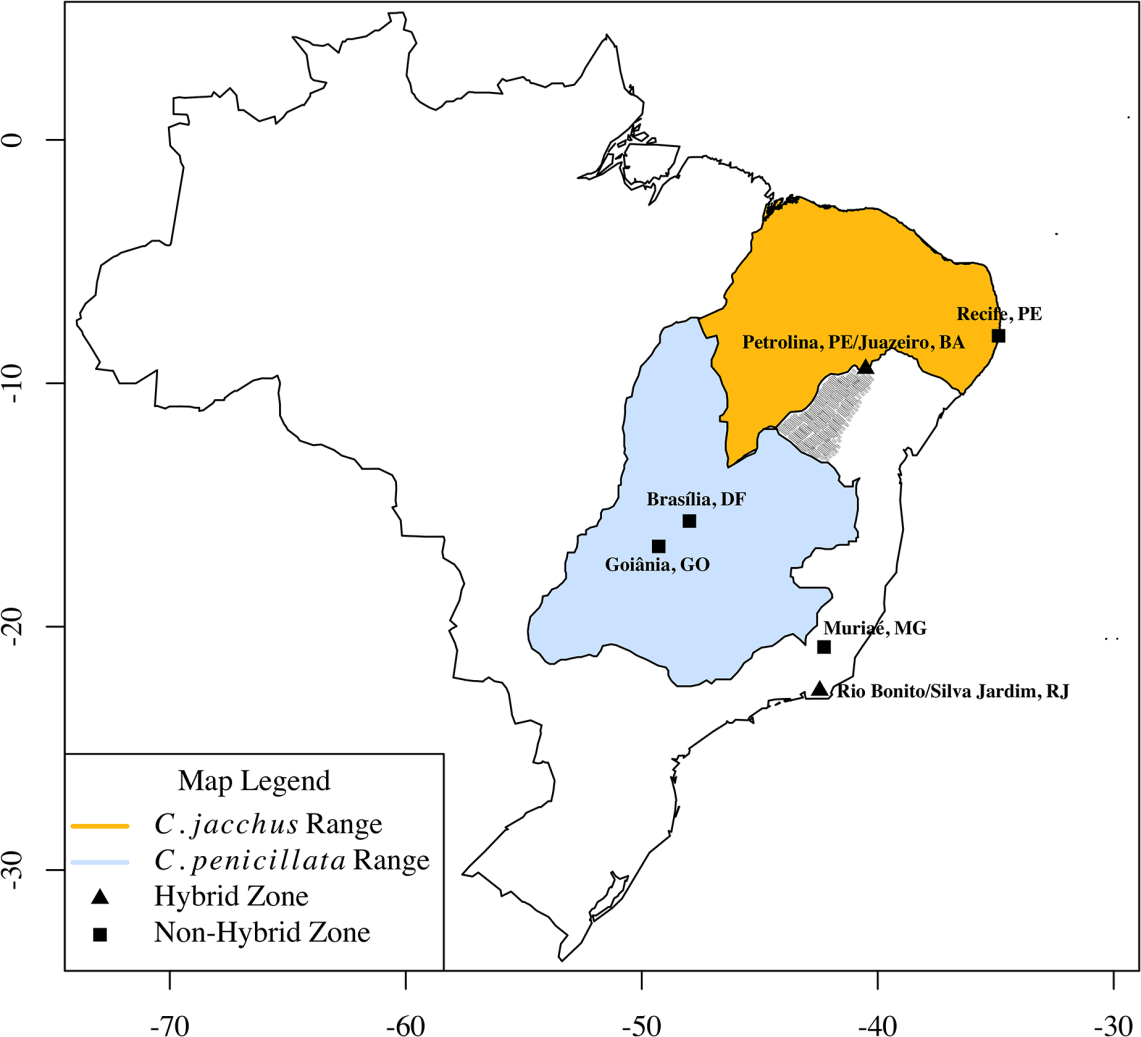
Brazilian *Callithrix jacchus* and *C*. *penicillata* ranges and sampling locations of parental and hybrid populations. Orange and light blue areas represent *C*. *jacchus* and *C*. *penicillata* ranges, respectively, based on 2014 IUCN Red List Spatial Data (http://www.iucnredlist.org/technical-documents/spatial-data). Thatched grey suggests region of *C*. *penicillata* presence based on Rylands *et al*. (1993, 2009) and our observations from this study. Degrees of longitude and latitude are, respectively, represented by the *x*- and the *y*-axes.

Between 2010 and 2011, biological samples were obtained from 80 *C*. *jacchus* and 44 *C*. *penicillata*, in both wild and captive (outbred) populations of each species. We also sampled 89 animals within two putative *C*. *jacchus* x *C*. *penicillata* hybrid zones. Phenotypic differences between the parental species and their hybrids are described in Malukiewicz *et al*. [23]. Locations of wild capture sites for pure and hybrid marmosets are shown in Fig 1. Sampling information is listed in Table 1 and latitude/longitude coordinates of the collection site for each individual are given in S1 Table. Additional *C*. *jacchus* samples were collected in 2005 and graciously donated by Dr. Maria Adélia Borstelmann de Oliveira (Table 1). This work included the collection of cheek swabs and skin samples from wild marmosets and cheek swabs, blood, and skin samples from captive marmosets. The Arizona State University Institutional Animal Care and Use Committee Animals approved the capture and sampling of both wild Brazilian and US captive marmoset populations (ASU IACUC, protocol #11-1150R). Permission for capture and tissue collection from wild marmosets was also obtained from the Brazilian National Council on the Development of Science and Technology (CNPq) and the Brazilian Ministry for the Environment and Natural Resources (IBAMA, protocol #28075–2). All possible steps were taken to minimize animal suffering and maximize their safety. Collection from captive animals was done opportunistically during routine procedures, following facility guidelines. Wild animals were captured with Tomahawk style traps under protocols developed by Drs. Boere and Ruiz-Miranda. Animals were anesthesized with ketamine. Detailed information about collection permits sample collection, storage, sampling sites/facilities, and DNA extraction from biological samples has been described in Malukiewicz et al. [23].

**Table 1:** Summary of sampled individuals of *C*. *penicillata* and *C*. *jacchus* from US captive and Brazilian wild pure populations and hybrid zones.

Populations	Type	Source	Year Collected	Individuals Sampled
*C*. *jacchus*	Captive	CRC^a^, Omaha, NE, US	2011	2 (2)
	Wild	IBAMA CETAS^b^, Recife, PE, Brazil	2011	24 (23)
	Captive	NEPRC^c^, Southborough, MA, US	2010	11 (9)
	Wild	Parque Dois Irmãos, and Tapacurá Reserve, PE, Brazil^d^	2005	43 (28)
*C*. *penicillata*	Captive	CRC^a^, Omaha, NE, US	2011	8 (7)
	Wild	Muriaé, MG; Brasila, DF; Goiânia, GO, Brazil	2011	28 (26)
	Captive	IBAMA CETAS^b^, Recife, PE, Brazil	2011	3 (3)
	Wild	IBAMA CETAS^b^, Goiânia, GO, Brazil	2011	5 (5)
*C*. *jacchus x C*. *penicillata* hybrids	Wild	Silva Jardim and Rio Bonito Municipalities, RJ, Brazil	2011	46 (45)
	Wild	Petrolina, PE and Juazeiro, BA, Brazil	2011	40 (40)
	Captive	CEMAFAUNA^e^, Petrolina, PE, Brazil	2011	3 (3)

Parentheses contain sample numbers with successful DNA extractions. Abbreviations within the table are footnoted below.

^a^Callitrichid Research Center, University of Nebraska at Omaha

^b^Wild Animal Triage Center, Brazilian Institute of the Environment and Natural Resources

^c^New England Primate Research Center

^d^Collected by Dr. Maria Adélia Borstelmann de Oliveira

^e^Center for Management of Fauna of the Caatinga

**Fig 2:**
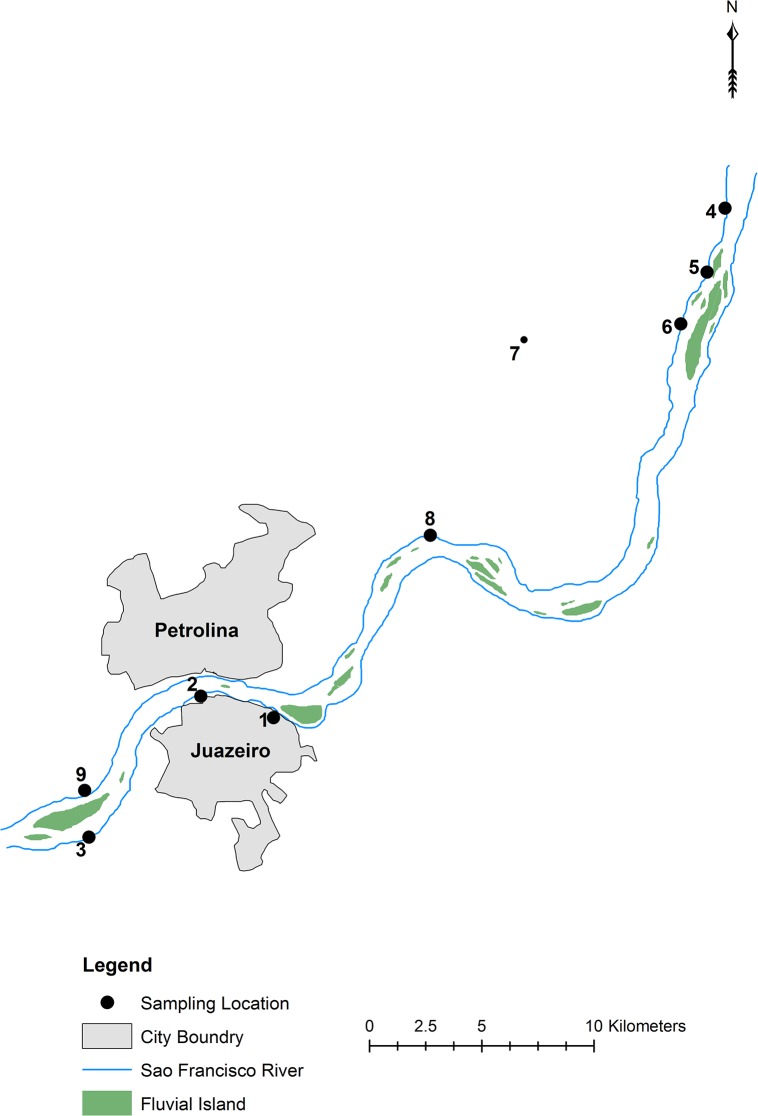
Detail of the Petrolina-Juazeiro natural hybrid zone. We sampled within the hybrid zone along an approximately 50 km transect paralleling the São Francisco River. Three sites were found to the south of the river: (1) Universidade do Estado da Bahia; (2) Chácara do Senhor Conrado dos Santos; and (3) Recanto do Sessego. Six sites were found to the north of the river: (4) Sítio Porto da Cruz; (5) Rio Verde; (6) Sítio Picos; (7) Sítio Carnaíba; (8) Chácara Galo da Briga; and (9) Chácara Bom Jesus.

The cities of Petrolina, Pernambuco (PE) and Juazeiro, Bahia (BA) make up a putative natural hybrid zone referred to as the “PJ natural zone” and a detailed view of capture locations within this zone is shown in Fig 2. The PJ natural zone lies at a species border between *C*. *jacchus* and *C*. *penicillata*, whose geographic ranges are separated by the São Francisco River with *C*. *jacchus* found to the north and *C*. *penicillata* found to the south. We consider this species contact zone an extension of the marmoset species ranges outlined in Rylands *et al*. [17].

**Fig 3:**
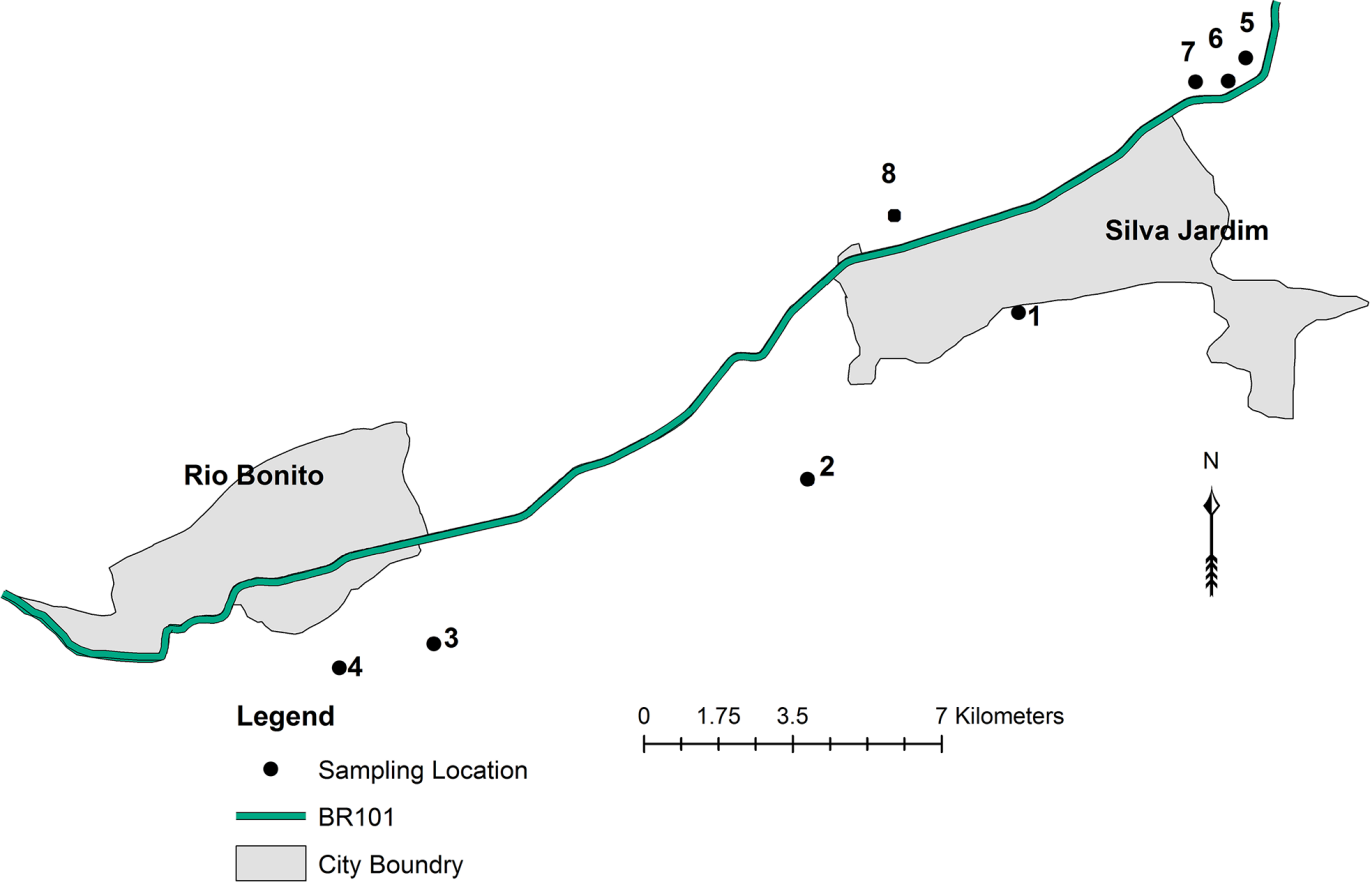
Detail of Rio de Janeiro State anthropogenic hybrid zone. We sampled within the zone along an approximately 30 km transect paralleling highway BR-101. Four sites were found to the south of the highway: (1) Boa Esperança; (2) House U; (3) Rio Vermelho I; and (4) Rio Vermelho II. Four sites were found to the north of the highway: (5) Fazenda dos Tamarins; (6) Pesque Pague; (7) Ponto do Camarão; and (8) Fazenda Afetiva.

The municipalities of Silva Jardim and Rio Bonito make up an anthropogenic hybrid zone in Rio de Janeiro state, abbreviated here as the “RJ anthropogenic zone.” Capture locations within the RJ anthropogenic zone are detailed in Fig 3. Marmosets in the RJ anthropogenic zone are descendants of introduced *C*. *jacchus* and *C*. *penicillata* present in the area since at least the mid-1980s [21]. The RJ anthropogenic zone is divided into northern and southern portions by a heavily used highway, BR-101.

### Marmoset Chimerism and Microsatellite loci

Marmoset chimerism (possession of at least two genomic lineages by a single individual) occurs as a result of frequent twinning in these primates and *in utero* exchange of stem cells by twins [24]. As a result, marmoset tissues can be derived from self and sibling embryonic cell lineages [25]. Ross *et al*. [25] showed that chimerism levels differ by tissue in *C*. *kuhlii*, with epithelial tissue having some of the lowest levels (12% chimeric) and blood-derived tissue having some of the highest levels (50% chimeric). We carried out a preliminary microsatellite genotyping analysis in *C*. *jacchus* using blood and epithelial tissues to determine whether the latter would be suitable for microsatellite genotyping of marmosets sampled during our study.

For this preliminary analysis, the tissue was donated by the New England Primate Research Center and DNA was obtained via a standard phenol-chloroform extraction protocol. We genotyped 10 unrelated individuals at two microsatellite loci, caja1 and caja5 [26]. The two loci were amplified in separate 24 uL polymerase chain reactions (PCR) using the AmpliTaq Gold PCR Kit with Buffer II (Life Technologies, USA) with the following reagents at final concentrations of 1X Buffer II, 0.8 mM total dNTPs, 1.5 mM MgCl2, 0.5 uM of each forward and reverse primer, and 0.025 U/uL Taq DNA polymerase. The thermocyclers settings on an MJ Research PTC-200 were as follows: (1) 94.5°C for 5 minutes, (2) 94.5°C for 45 sec, (3) 48°C for 30 seconds for caja5 or 55°C for 30 seconds for caja1, (4) 72°C for 30 seconds, (5) repeat steps 2–4 a total of 36 times for caja5 or 35 times for caja1, and (6) 72°C for 1:30 minutes. Most side-by-side comparisons of electrophoretograms of microsatellites amplified from blood and skin samples of the same individual were consistent with each other, though blood samples occasionally showed 3 allele genotypes when skin genotypes showed 2 alleles. Thus, we only used epithelial tissues from wild and captive marmosets that were part of our main dataset.

We incorporated caja1 and caja5 into a larger panel as recommended by Vaha and Primmer [27] to differentiate between hybrid and pure individuals. For the larger microsatellite panel, we tested a total of 50 dinucleotide markers developed for marmosets and lion tamarins [26, 28–30]. Six loci were excluded due to poor amplification, and the remaining 44 loci were polymorphic in 62 *C*. *jacchus* and 41 *C*. *penicillata*. These 44 loci were amplified in 15 multiplex reactions (S2 Table), each at a 10 uL volume, using the Qiagen Multiplex PCR Kit following manufacturer directions at a modified annealing temperature of 64°C. Forward primers for each locus were labeled with a fluorescent dye. All loci were subject to fragment analysis on an ABI 3730 sequencer with GeneScan 500 LIZ (Life Technologies, Carlsbad, CA, USA) size standard at the Arizona State University DNA Core Laboratory (Tempe, AZ, USA). Alleles sizes were scored using GENEMARKER (Softgenetics) and checked manually.

Microsatellite genotypes were placed into a data matrix following the GENEPOP [31] format that was organized by sampled individual and population (S1 Data). Missing genotypes within the data matrix were flagged as “000000,” but these missing data were left untreated in downstream analyses. Hale *et al*. [32] indicate that data are needed from 25–30 individuals per population for accurate population genetic analyses based on allele frequencies. Because we had at least 29 genotyped individuals per locus for each population, missing data were not expected to strongly influence our allele frequency estimates at each microsatellite locus.

### Data analysis

Locus allele frequencies for the two parental species and within hybrid zones were calculated with GENEPOP 4.2 [31]. The “exact test” [33, 34, 35] in GENEPOP was carried out to test each locus within each species and hybrid zone for deviation from Hardy-Weinberg equilibrium (HWE) using the MCMC method with 10,000 dememorization steps, 1000 batches, and 10,000 iterations per batch. The same software was used to test pairwise linkage disequilibrium (LD) within each species and hybrid zone under the same MCMC settings as for HWE. *P*-values for LD and HWE tests were adjusted with the Bonferroni correction for multiple comparisons [36]. FSTAT 2.9.3.2 [37] was used to determine the number of observed alleles, allelic richness (R), and F_IS_ [38] for each locus within the two species and hybrid zones. Observed (H_O_) and expected heterozygosity (H_E_) were determined with GENODIVE [39].

Possible presence of null alleles within the dataset was examined by MICROCHECKER [40], which also checks for other genotyping errors such as stuttering and short allele dominance. As our dataset likely contains null alleles (see Results), we calculated locus null allele frequency (r) within each population using 10,000 iterations of the EM algorithm [41] as implemented in the program FREENA [42, 43]. Chapuis and Estoup [42] found the EM method to be the best r estimator among three commonly used estimators. Additionally, these authors found only a weak effect of null alleles on H_E_ across a large range of r. Thus, we did not make corrections for any of the within-population analyses discussed above.

We next examined population differentiation between the two study species as well as subpopulations found on the respective northern and southern sides of each hybrid zone. FREENA can calculate F_ST_ values that are corrected and unbiased for null alleles [42], and we used this software to obtain uncorrected and corrected values of the statistic. The corrected F_ST_ value is based on Weir [34] and includes only visible allele sizes. Statistical population differentiation analysis was also carried out in GENODIVE using AMOVA R_ST_ statistics, based on the stepwise mutation model for microsatellites, and 10,000 permutations.

We applied two complementary Bayesian clustering approaches to determine levels of *C*. *jacchus* and *C*. *penicillata* admixture, STRUCTURE 2.3.4 [44] and BAPS 6.0 [45–47], within the RJ and PJ hybrid zones. Our decision to use both clustering programs was based on Bohling *et al*. [48], who found that while STRUCTURE was more successful at identifying hybrid individuals and calculating their admixture levels, BAPS was less likely to misclassify pure individuals as hybrids. Genotypes of pure species, in captive and wild populations found outside of hybrid zones, were used with both methods as reference samples upon which cluster allele frequencies were estimated. The reference samples also aided in ancestry estimation and identification of hybrids among individuals sampled within the two hybrid zones. Adapting the approach of Godinho *et al*. [49], we made the *a priori* assumption that the number of clusters (K) is two, i.e. that there are two ancestral populations contributing to the gene pool in either hybrid zone. Based upon the simulation studies of Vaha and Primmer [27], we considered an individual a hybrid if 0.10< *q* <0.90 for that individual (the fraction of the individual’s genome inherited from population *k*).

We set up 10 independent runs in STRUCTURE under the USEPOPINFO model to allow for use of pre-defined parental species groups to aid classification of hybrid samples following conditions per run with MIGRPRIOR = 0.05 (default value). *Callithrix penicillata* individuals were indicated by Popflag = 1, *C*. *jacchus* individuals were identified by Popflag = 2, and all individuals sampled from hybrid zones were identified by Popflag = 0. The PFROMPOPFLAGONLY option was turned on for allele frequency estimation only based on the parental species. Admixture levels of hybrids were estimated for hybrid zone individuals under the admixture ancestry model, which assumes that some fraction of an individual’s genome comes from the two parental clusters. This model was used with the default setting of an inferred alpha initially set to 1.0. The default correlated allele frequency model was used for all runs, and each run consisted of 80,000 burn-in steps followed by 8,000,000 MCMC iterations. The ten runs were checked for consistency in summary statistics and convergence of parameter values. Average *q*-values for hybrid samples across the independent runs were determined with the full search algorithm of CLUMPP 1.1.2 [50]. DISTRUCT 1.1 [51] was used to produce a graphical display of hybrid zone admixture as determined by STRUCTURE. Null allele corrections were not applied during STRUCTURE analyses, as Carlsson [52] found that the presence of null alleles at microsatellite loci “would probably not alter the overall outcome of assignment testing.”

Second, we used BAPS by conducting an admixture analysis based on pre-defined *C*. *jacchus* and *C*. *penicillata* clusters of individuals from outside of marmoset hybrid zones. Genotypes of pure individuals were used to define allele frequencies. Each BAPS run was executed with the following conditions: minimum population size was set to the default size of 5, there were 300 iterations per run, 200 reference individuals, and the number of iterations for admixture estimations of reference individuals was set at 10. Five independent BAPS runs were carried out and consistency of ancestry estimation for hybrid zone samples was verified between run replicates. Null allele corrections were not applied to BAPS analyses, as simulations studies by Chapuis *et al*. [53] found that assignment results for this software actually improve in the presence of null alleles at microsatellite loci.

Because the assumed priors and efficiency, or proportion of correctly identified hybrids out of a total number of actual hybrids in a sample [27], of the above analyses cannot be assessed statistically, we conducted simulation studies to evaluate the power of our reference dataset to detect hybrids and to estimate *q*. We simulated 10 STRUCTURE datasets and 5 BAPS datasets composed of all reference samples, 10 F1 hybrids, 10 F2 hybrids, 10 *C*. *jacchus* x F1 backcross hybrids, and 10 *C*. *penicillata* x F1 backcross hybrids. All simulated hybrid classes were created with HYBRIDLAB 1.0 [54] and analyzed with STRUCTURE and BAPS as described for actual data sets. Efficiency was then calculated as in Vaha and Primmer [27].

We also employed one non-Bayesian method to study the partition amongst hybrid and pure groups by using GENODIVE to carry out a principal component analysis (PCA). The PCA used a covariance matrix based on individual multilocus genotypes as presented in S1 Data. We used the first and second components of the PCA to summarize differences in multilocus genotypes data among the four population groups as a final analysis of population clustering of pure species and hybrid zone samples.

## Results

### Deviations from Hardy-Weinberg equilibrium and linkage disequilibria

Deviations from HWE varied among loci within the parental species and the hybrid zones (S3–S6 Tables). For parental species, 20 out of 44 *C*. *penicillata* loci and 5 out of 44 *C*. *jacchus* loci were in LD. MICROCHECKER indicated the possible presence of null alleles for almost all loci out of HWE in *C*. *penicillata*, but within-species pooling of samples from isolated captive and geographical subpopulations may have also caused loci to be out of HWE (Walhund effect). Null allele presence indicated by MICROCHECKER and Walhund effect for *C*. *jacchus* loci may also explain some of the significant heterozygote deficiencies. In the hybrid zones, 12 out of 44 loci in the PJ natural zone and 6 out of 44 loci in the RJ anthropogenic zone were in LD. All loci out of HWE had positive F_IS_ values indicating heterozygote deficiencies in pure and hybrid populations. MICROCHECKER flagged most hybrid zone loci not in HWE for the likely presence of null alleles. For the PJ natural zone, pooling of samples from two parapatric and genetically differentiated forms separated by a putative dispersal barrier (the São Francisco River) may explain part of the observed significant heterozygote deficiency. In the RJ anthropogenic zone, pooling of several subpopulations separated by highway BR-101, also a putative dispersal barrier, may explain observed Hardy-Weinberg disequilibrium.

The majority of markers used in this study map to different chromosomes of the common marmoset published genome (calJac 3 build, genome.ucsc.edu), and those located on the same chromosome are expected to be far enough apart to minimize the chance of physical linkage within the *C*. *jacchus* and *C*. *penicillata* genetic backgrounds. Nonetheless, significant LD was found in some pairwise locus comparisons out of a total of 946 comparisons within each group as follows: (1) pairs of markers where each locus was found on a different chromosome totaled 29 in *C*. *penicillata*, 8 in *C*. *jacchus*, 34 in the PJ natural zone, and 14 in the RJ anthropogenic zone, (2) and marker pairs with both loci located on the same chromosome totaled 1 in *C*. *penicillata*, 3 in *C*. *jacchus*, 3 in the PJ natural zone, and 11 in the RJ anthropogenic zone. Demographic factors such as population structure may help explain inflated levels of LD, particularly in the case of *C*. *jacchus* and *C*. *penicillata* where we pooled samples from different captive and geographic origins. For the RJ anthropogenic zone, the high number of physically linked loci in disequilibrium may indicate that they continue to segregate together within their ancestral genetic background if the founder populations originally possessed limited allelic diversity at these loci.

### Allele frequencies, genetic diversity, and population differentiation

An averaged summary of various genetic diversity measures for pure and hybrid groups is shown in Table 2 and expanded for individual loci in S3–S6 Tables. *C*. *penicillata* generally exhibited higher allele numbers (mean number of alleles per locus = 10.864) and allelic richness (mean allelic richness = 0.276) at these microsatellite loci than *C*. *jacchus* (mean number of alleles per locus = 8.386, mean allelic richness = 7.120). H_E_ levels were broadly similar between the two species, but for many *C*. *penicillata* loci H_O_ levels were lower than expected, while H_E_ and H_O_ were similar for *C*. *jacchus*. Measures of genetic diversity in the PJ natural zone were similar to values seen in the parental species. The number of observed alleles and allelic richness within the RJ anthropogenic zone was the lowest out of the four groups, but H_E_ end H_O_ were comparable to the other three groups. Loci with lower H_O_ than H_E_ are loci with a significant heterozygote deficit and those that may contain null alleles. Thus allele drop-out may have caused deflation of H_O_ relative to H_E_ at such loci, especially in *C*. *penicillata*.

**Table 2:** Averages of various genetic diversity indices for *C*. *penicillata* and *C*. *jacchus* and hybrid groups.

Group	N	*A*	R	r	H_o_	H_E_	F_IS_
***C*. *penicillata***	37.84	10.86	10.28	0.09	0.62	0.80	0.22
***C*. *jacchus***	55.57	8.39	7.12	0.04	0.62	0.67	0.08
**PJ Hybrid Zone**	39.39	8.30	7.71	0.07	0.58	0.70	0.17
**RJ Hybrid Zone**	41.30	6.75	6.43	0.05	0.63	0.72	0.13

N is number of individuals sampled at a locus, A is the number of alleles at a locus, R is allelic richness, r is EM null allele frequency, Ho is observed heterozygosity, H_E_ is expected heterozygosity, F_IS_ is the inbreeding coefficient.

Allele frequencies uncorrected and corrected for null allele presence across the 44 microsatellite loci are shown in S7 Table for parental and hybrid populations. No true fixed diagnostic loci between *C*. *jacchus* and *C*. *penicillata* were found. *Callithrix penicillata* had on average 4.84 (or 44.6%) private alleles per locus and *C*. *jacchus* had 2.65 (or 31.6%) private alleles per locus. Most private alleles in *C*. *penicillata* were found together in along a continuous range of sizes. *C*. *jacchus* private alleles were found as intermittent singletons among non-private alleles within a continuous range of shared allele sizes. The remaining alleles overlapped in the two species but allele frequencies differed interspecifically. Alleles present in the hybrid zones were a mosaic of those alleles found in the parental population, but the PJ natural zone contained a much larger representative sample of parental alleles than did the RJ anthropogenic zone. The PJ natural zone had an average of 1.52 (or 18.3%) private alleles per locus, mostly as singletons and the RJ anthropogenic zone had an average of 1.36 (or 20.1%) private alleles per locus, also mostly singletons.

F_ST_ values corrected and uncorrected for null allele presence were similar for each pairwise comparison (Table 3). *Callithrix jacchus* and *C*. *penicillata* showed a moderate level of differentiation (uncorrected F_ST_ = 0.183). AMOVA analysis revealed that a significant portion (*P*-value = 0.000) of genetic variation occurred at the species level (32% R_ST_-based). The northern and southern sides of the respective hybrid zones also showed significant pairwise levels of genetic variation (*P*-value = 0.000) at a similar level observed for parental populations. AMOVA indicated that 26% of variation in the PJ natural zone was found between northern and southern subpopulations (*P*-value = 0.000, R_ST_-based). In the RJ anthropogenic zone, 14% of genetic variation was found between populations separated by highway BR-101 (*P*-value = 0.000, R_ST_-based).

**Table 3:** Pair-wise F_ST_ indices.

Pair-Wise Comparison	^U^F_ST_	^C^F_ST_
*C*. *penicillata- C*. *jacchus*	0.18	0.17
PJ N- PJ S	0.20	0.19
RJ N- RJ S	0.17	0.17

N and S indicate northern and southern portions of each hybrid zone. U and C indicate values uncorrected and corrected for null alleles, respectively.

**Fig 4:**
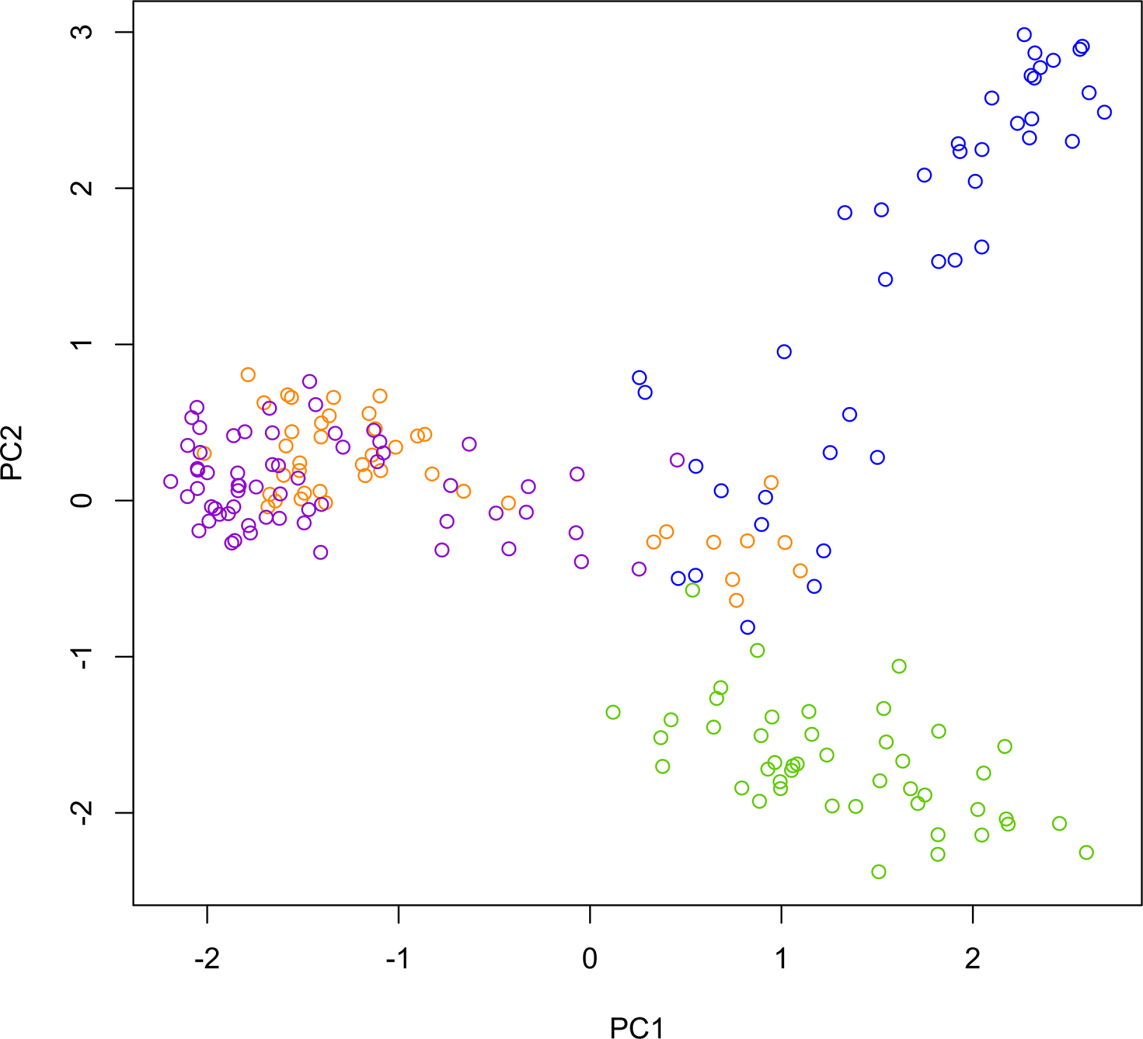
PCA of microsatellite allele frequencies. Plot of first and second components from the PCA showing genetic differences in terms of microsatellite multilocus genotypes between marmoset parental species and hybrid zone samples. Individual *C*. *jacchus* are colored in green, individual *C*. *penicillata* are colored in purple, and individuals from the PJ and RJ hybrid zones, respectively, are colored blue and orange.

In the PCA (Fig 4 and Table 4), the first and second components accounted for 19.41% of the total variation of multilocus genotypes. A bivariate plot shows that the first and second components defined differences between *C*. *jacchus* and *C*. *penicillata*. On the other hand, high genotypic similarity between *C*. *jacchus* and PJ natural hybrid zone marmosets was observed along the first component of the bivariate plot. The RJ anthropogenic hybrid zone population was very similar to *C*. *penicillata*, especially along the second component.

**Table 4:** Eigenvalues from PCA of genetic variation between parental species and populations from hybrid zones.

Principal Component	Eigenvalue	%Variance	Cumulative
1	2.40	11.76	11.76
2	1.56	7.65	19.41

### Hybrid zone admixture patterns

**Fig 5:**
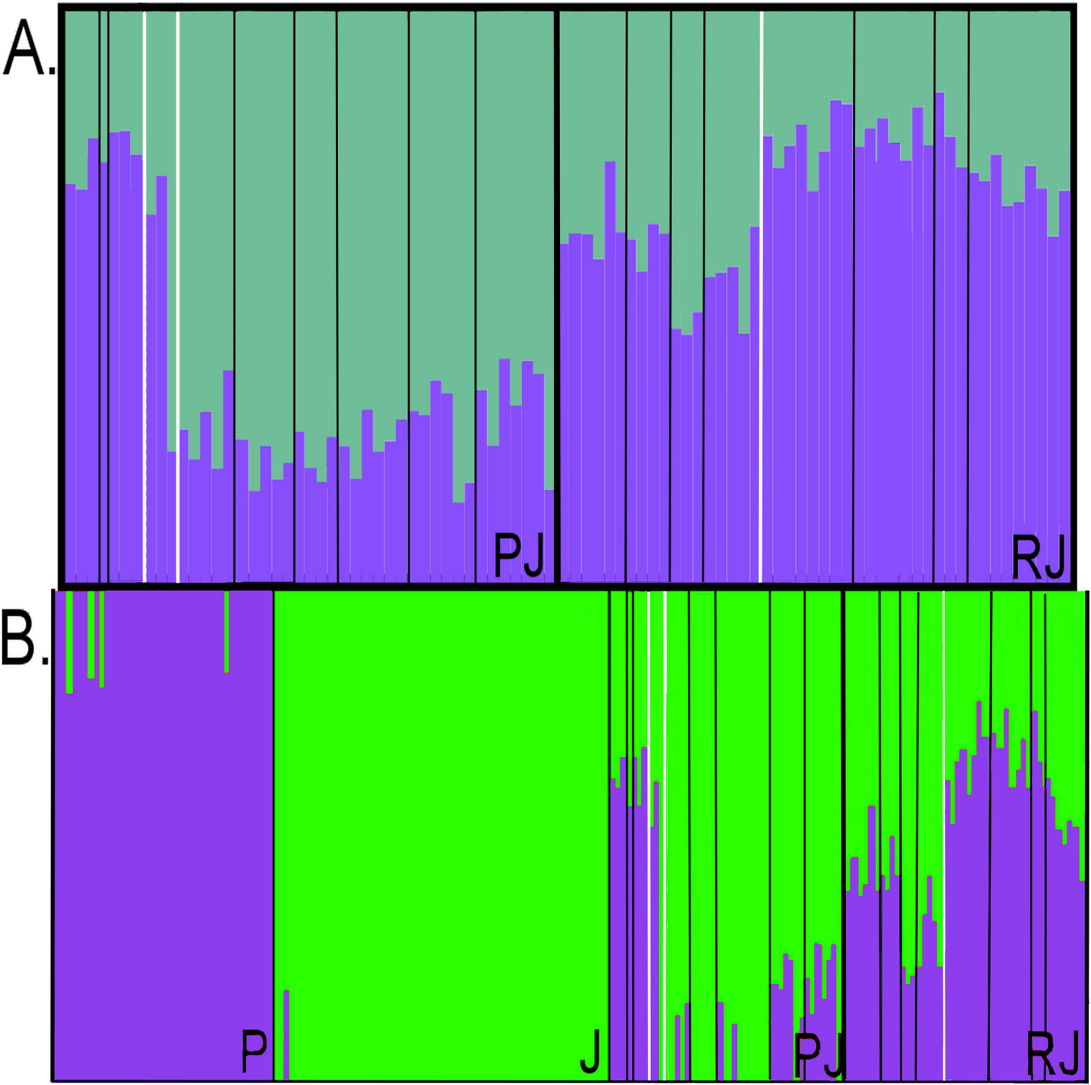
Admixture plots resulting from cluster analysis. A) Plots of *C*. *jacchus* and *C*. *penicillata* admixture within the two hybrid zones as assigned by STRUCTURE. The two hybrid zones are labeled by their initials. B) Plots of *C*. *jacchus* and *C*. *penicillata* BAPS admixture probabilities. Plots are divided by reference *C*. *penicillata* individuals (P), reference *C*. *jacchus* (J), and the hybrid zones labeled by their initials. In both plots, purple and green bar proportions indicate *C*. *penicillata* and *C*. *jacchus* ancestry, respectively. Within the PJ and RJ panels, black lines separate individual capture sites within each hybrid zone, following the order given in Fig 2 for the PJ zone and Fig 3 for the RJ zone. White lines within each hybrid zone panel separate the southern and northern portions of each hybrid zone. Also, in panel A, CEMAFAUNA captive marmosets are found between the two white lines.

For all cluster analyses, admixture coefficients are relative to *C*. *penicillata* ancestry with *q* = 1.0 indicating full ancestry and *q* = 0.0 indicating no *C*. *penicillata* ancestry. All runs conducted in STRUCTURE were concordant for *C*. *jacchus* and *C*. *penicillata* admixture levels in hybrid individuals, and results averaged across 10 runs by CLUMPP are shown in Fig 5A. Please note that STRUCTURE did not calculate *q*-values for the parental species reference samples, so those individuals were not included in the plot shown in Fig 5A. The 90% confidence intervals for *q*-values of hybrid individuals are shown in S1 Fig. Within both the PJ natural zone and RJ anthropogenic zone, all sampled individuals had admixture coefficients within the range of 0.10<*q*<0.90, indicating a strong possibility that most of these marmosets were *C*. *penicillata* x *C*. *jacchus* hybrids. The average admixture coefficient for the entire PJ natural zone was *q* = 0.36, ranging from 0.14 to 0.79. Marmosets showed on average *q* = 0.75 on the south side of the river within the PJ natural zone, and an average of *q* = 0.26 on the north side of the river. Thus, this indicated that the former possess mostly ancestry from *C*. *penicillata* and the latter possess ancestry mostly from *C*. *jacchus*. However, these data also strongly suggested bi-directional gene flow and introgression of *C*. *jacchus* and *C*. *penicillata* across the PJ natural zone. Ten PJ natural zone individuals had 90% *q*-value confidence intervals that overlapped with the *q*-values indicative of non-admixed individuals. More specifically, the *q*-value confidence intervals of 7 marmosets sampled north of the river dropped into range of full *C*. *jacchus* ancestry and 3 marmosets sampled south of the river dropped within range of full *C*. *penicillata* ancestry. The average admixture for marmosets in the RJ anthropogenic zone was *q* = 0.69, with a range of 0.53 to 0.86. Average *q*-values on the north side of this zone were 0.75 and on the south side average values were 0.60, thus overall, ancestry on both sides of the RJ anthropogenic zone tended more towards *C*. *penicillata* than *C*. *jacchus*. Nine RJ anthropogenic zone marmosets, all from the northern side, showed 90% confidence interval ranges that fell into the range of *q*-values expected for pure *C*. *penicillata*.

Admixture coefficients calculated by BAPS were concordant between replicate runs, but BAPS coefficient values for all hybrid zone individuals (Fig 5B) were lower than those calculated by STRUCTURE. BAPS (unlike STRUCTURE) calculated admixture probabilities for reference individuals in addition to putative hybrid individuals. A visual inspection of Fig 5B highlights the higher level of admixture within hybrid zones than outside of hybrid zones. BAPS considered 19 individuals within the PJ natural zone as pure *C*. *jacchus*. The average BAPS coefficient in the PJ natural zone was *q* = 0.20, ranging from 0 to 0.68, and the average RJ anthropogenic zone BAPS coefficient was *q* = 0.53, with a range from 0.31 to 0.77. As with STRUCTURE estimates for the north PJ side, ancestry tended towards *C*. *jacchus* (average *q* = 0.09) and on the south side ancestry was biased towards *C*. *penicillata* (average *q* = 0.63). BAPS *q*-values, in contrast to STRUCTURE, indicated pure *C*. *jacchus* ancestry for 18 individuals on the north PJ natural zone side. Overall, BAPS admixture analysis again suggested bidirectional gene flow and introgression between *C*. *jacchus* and *C*. *penicillata* across the PJ natural zone. The average north-side RJ anthropogenic zone BAPS *q*-value was 0.61, while the south-side average was 0.41, a trend concordant with that of STRUCTURE *q*-values. No marmosets within the RJ hybrid zone were of pure *C*. *jacchus* or *C*. *penicillata* ancestry according to BAPS *q*-values.

### Simulation results

Simulation files contained the full set of pure *C*. *jacchus* and *C*. *penicillata* multilocus genotypes, plus 10 each of F1 hybrids, F2, hybrids, and backcross hybrids for each species. S8 Table shows summary information across 10 simulated STRUCTURE runs, averaged across 10 replicate runs each. These data showed that STRUCTURE classified all simulated hybrids with 100% efficiency at the 0.9 *q*-value threshold. Further, 90% confidence intervals for *C*. *jacchus* and *C*. *penicillata* backcross hybrids stayed within our *q* = 0.90 threshold for hybrid classification. Thus our dataset had 100% power to detect F1, F2, and backcross hybrids of both species using the STRUCTURE clustering algorithm. S9 Table shows a summary of *q*-values and *q*-value ranges for simulated BAPS runs averaged across 5 repetitions per simulated dataset. BAPS showed a 100% efficiency of correct hybrid assignment for F1, F2 and *C*. *jacchus* backcross hybrids. BAPS calculated full *C*. *penicillata* ancestry for 1 out of 10 *C*. *penicillata* backcross hybrids in 2 out of 5 datasets, giving it an average of 96% efficiency of correct assignment for this hybrid class. STRUCTURE *q*-values tended to be higher than BAPS *q*-values. However, our reference data set of pure individuals allowed both programs to estimate, on average, appropriate admixture values expected for F1, F2, and backcross hybrids. STRUCTURE and BAPS estimated admixture levels within the PJ and RJ hybrid zones were proportionally similar to simulated early and later generation hybrids.

## Discussion

### Differentiation between *C*. *jacchus* and *C*. *penicillata* based on autosomal data

We genetically characterized *C*. *penicillata* and *C*. *jacchus* interbreeding at a natural and anthropogenic hybrid zone using a large panel of 44 autosomal microsatellite loci and reference samples from both parental species. While the reference dataset did not contain any fixed species-specific alleles at any locus between the two study species, there were allele frequency differences between species and each species possessed private alleles. Given the very recent divergence date estimated by Perelman *et al*. [15] for *C*. *penicillata* and *C*. *jacchus*, diagnostic loci between the species may emerge with time. Our microsatellite panel combined markers from several previous studies of wild and captive *C*. *jacchus* (e.g. [26, 28, 30]). There have not been any published microsatellite data for *C*. *penicillata*. Thus, this combined marker set enabled us to expand previous reports of *C*. *jacchus* genetic diversity and give an initial report of *C*. *penicillata* genetic diversity by using a large number of microsatellite loci in a large number of samples from both species. Averages at microsatellite loci were slightly higher in *C*. *penicillata* in than *C*. *jacchus* for the number of total observed alleles, private allele number, allelic richness, and expected heterozygosity. Similar differences in diversity between the two species were also observed at the mtDNA CR as described in Malukiewicz *et al*. [23]. Nonetheless, if our reference samples covered a larger area of the *C*. *jacchus* range, reported genetic diversity indices for that species may have been closer to that reported for *C*. *penicillata*. We found evidence for moderate levels of differentiation in the parental species using various population genetics and clustering techniques, and post-hoc simulations of the reference dataset indicated it is powerful enough to detect hybrids with varied levels of admixture.

### Genetic diversity and admixture within hybrid zones

PJ and RJ hybrid zone genetic diversity indices based on autosomal markers were broadly similar to those found in the two parental species. Nonetheless, cluster analyses as well as PCA allowed for a deeper insight into admixture within the two hybrid zones. The general observed geographical distribution of admixture patterns within the PJ natural zone matched the geographical boundary of *C*. *jacchus* to the north of the São Francisco River and *C*. *penicillata* to the south [17]. Observed admixture patterns and PCA groupings also suggested that northern subpopulations receive more gene flow from parental *C*. *jacchus*, and subpopulations to the south exchange genes mostly with parental *C*. *penicillata* populations. Thus, this pattern suggested an important evolutionary role for the São Francisco River as a barrier to gene flow between *C*. *jacchus* and *C*. *penicillata*.

**Fig 6:**
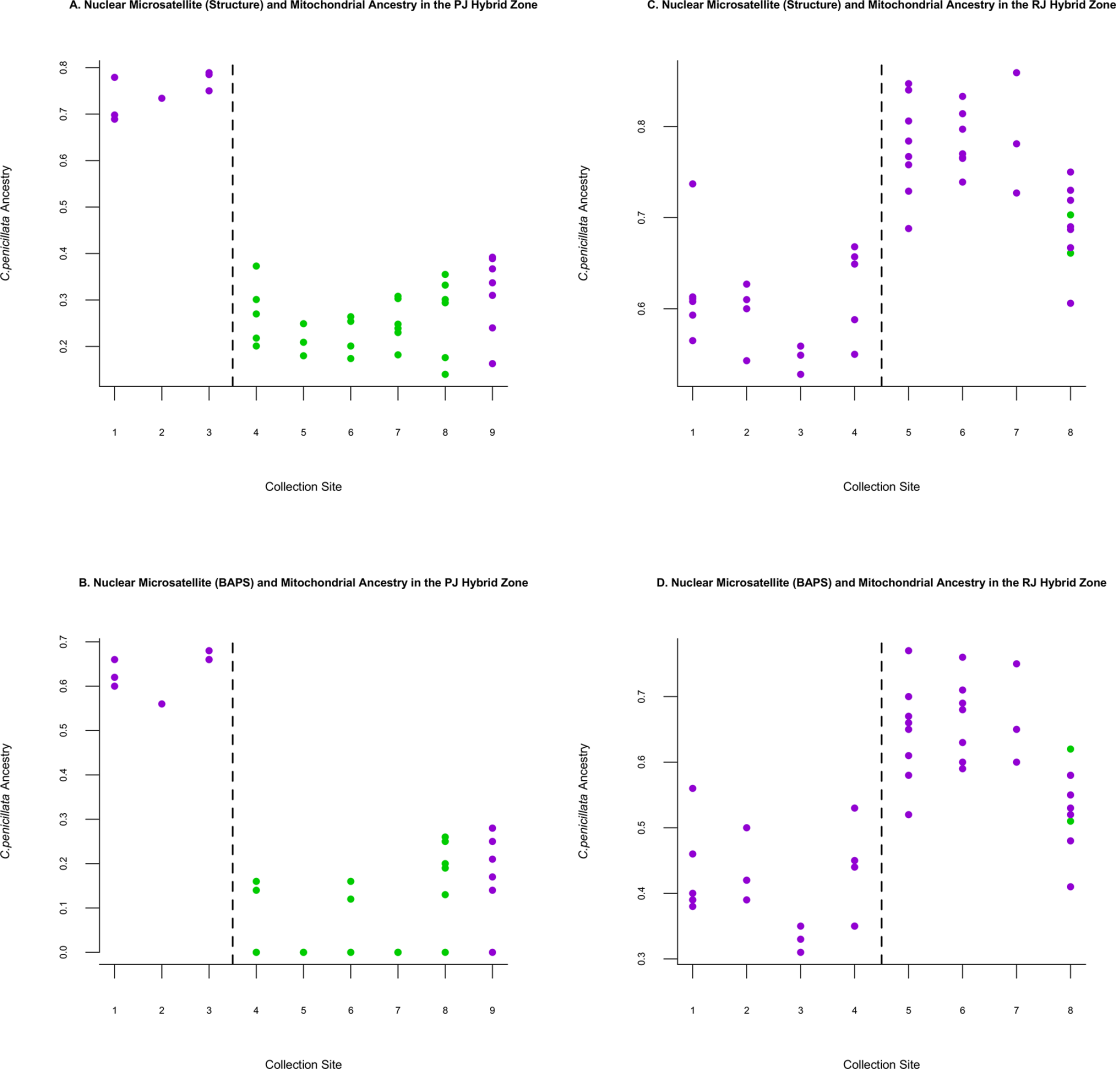
Combined results from microsatellite cluster analysis and mtDNA data from Malukiewicz *et al*. [23]. Panels A-D are separated by the PJ natural hybrid zone/RJ anthropogenic hybrid zone and STRUCTURE/ BAPS microsatellite *q*-values and coupled with mtDNA haplotypes from Malukiewicz *et al*. [23]. The *x*-axis shows individual PJ and RJ collection sites, which are numbered the same as in Figs 2 and 3. The *y*-axis indicates the level of *C*. *penicillata* ancestry. The dashed lines indicate a south/north hybrid zone divide in each figure panel. Purple indicates *C*. *penicillata* mtDNA haplotypes and green indicates *C*. *jacchus* mtDNA haplotypes.

However, both our autosomal and the mitochondrial data from Malukiewicz *et al*. [23] suggested that the São Francisco River may indeed be a leaky barrier for marmoset gene flow to allow genetic introgression between *C*. *jacchus* and *C*. *penicillata*; albeit our autosomal data showed two-way genetic introgression and the mtDNA data showed one-way introgression across the PJ natural hybrid zone (see Fig 6). Specifically, introgression of *C*. *penicillata* mtDNA into *C*. *jacchus* was localized to a single sampling site in the PJ natural zone [23] (Chácara Bom Jesus, site 9 in Fig 6). In contrast, two-way introgression was indicated by the current autosomal microsatellite data since cluster analyses identified genetic input from *C*. *jacchus* and *C*. *penicillata* along both banks of the São Francisco River (Fig 6). It is thought that fluvial islands facilitate gene flow between marmosets on either side of the river as it reshapes these islands over time and occasionally allows them to connect with each riverbank (personal observation, LCMP). Coincidently, the Sítio Porto da Cruz (Fig 6A and 6B site number 4) and Chácara Bom Jesus (Fig 6A and 6B site number 9) sampling sites showed among the highest autosomal components of *C*. *penicillata* ancestry for marmosets sampled on the northern side of the PJ natural zone. Both sites are found within close proximity to large São Francisco River fluvial islands. Thus, both our autosomal data and Malukiewicz *et al*. [23] showed evidence favoring fluvial islands as corridors for marmoset gene flow between the northern and southern banks of the São Francisco River.

Cluster admixture coefficients showed that individuals sampled on the southern side the PJ natural zone had mostly *C*. *penicillata* ancestry. However, the majority of our PJ natural zone samples came from the northern side of the zone (largely the result of limited access to sampling sites on privately held farms on the south side), and there was a dominant *C*. *jacchus* ancestry component in these individuals. Grouping patterns within the PCA corroborated our PJ natural zone cluster data. Since our genetic sampling within the PJ natural zone focused primarily on the *C*. *jacchus* north side of the São Francisco River, we most likely overrepresented the *C*. *jacchus* ancestry component within the PJ natural zone as a result of an unintended sampling bias. Most likely, an extended sampling along the south bank of the São Francisco River would support the observed pattern of majority *C*. *penicillata* autosomal ancestry for marmosets found on that side of the PJ natural zone.

Analyses of the RJ artificial hybrid zone indicated more intermediate levels of *C*. *jacchus* and *C*. *penicillata* admixture, with a bias towards *C*. *penicillata* ancestry. The RJ anthropogenic zone contained, on average, a somewhat lower number of alleles and allelic richness than that observed in either parental population, possibly a consequence of the lack of direct gene flow between this zone and parental populations. PCA and cluster analysis indicated that the ancestry of marmosets found within the RJ anthropogenic zone was closer to *C*. *penicillata* than *C*. *jacchus*. Surprisingly, heterozygosity levels within the RJ anthropogenic zone were comparable to those of parental populations, despite the isolated location of this zone in relation to the natural distribution of *C*. *jacchus* and *C*. *penicillata*. Malukiewicz *et al*. [23] also showed a stronger mitochondrial component of *C*. *penicillata* ancestry than that of *C*. *jacchus* within the RJ artificial hybrid zone (Fig 6).

It is sometimes assumed that populations of introduced animals are depauperate of genetic variation due to the effect of drift on small founder populations, but multiple introductions can boost genetic diversity in introduced populations [55, 56]. Given that three different mtDNA D-loop haplotypes (2 *C*. *penicillata* and 1 *C*. *jacchus*) were found found in the RJ artificial hybrid zone [23] and the high heterozygosity at microsatellitle loci we demonstrated here, it is possible that multiple introductions of both parental species occurred in the RJ anthropogenic hybrid zone. However, it is plausible that since these introductions mtDNA diversity within the RJ anthropogenic zone may have diminished due to genetic drift, as mtDNA has a smaller effective population size than nuclear DNA [57]. On the other hand, autosomal loci may still be buffered from drift due to their larger effective population size, but with time we might also expect heterozygosity levels at autosomal loci to decrease in the RJ anthropogenic zone. How fast this may occur is not clear, but marmoset populations within this zone tend to be isolated from one another amongst forest fragments and are probably cut off from gene flow between fragments as well as from parental populations (personal observation CRRM, JM). This level of isolation may threaten future levels of genetic diversity among RJ anthropogenic zone subpopulations with increasing levels of inbreeding.

### Implications for marmoset evolution, conservation, and future research

Genetic signatures of hybridization can vary for each hybrid zone as evolutionary and ecological dynamics can also differ between any set of interbreeding taxa. Bimodal hybrid zones at geographical contact points between different populations contain individuals that are genotypically similar to one or the other parental taxa with few intermediates, usually requiring evidence of strong LD and indicating strong assortative mating and pre-zygotic reproduction barriers [58]. Intermediate genotypes dominate in a unimodal contact hybrid zone where assortative mating is not as strong [58]. In the PJ natural zone, ancestry can be bimodally classified as either mostly *C*. *jacchus* or *C*. *penicillata*, whereas the highly admixed subpopulations of the RJ anthropogenic zone possess a more unimodal genotype distribution. Compared to the RJ anthropogenic zone, we also observed much higher levels of Hardy-Weinberg disequilibrium and LD within the PJ natural zone. The conditions observed within the PJ natural zone are similar to what Arias *et al*. [59] observed in a *Heliconius erato venus* and *Heliconius erato chestertonii* butterfly hybrid zone, which the authors presented as strong evidence of incipient speciation in these taxa. Reproductive isolation between various *Callithrix* species is not complete [16], but the São Francisco River seems to be an important geographical, albeit porous, reproductive barrier in the PJ natural zone that promotes assortative mating within the two distinct lineages found on the northern and southern river banks.

Natural geographical barriers are probably important to overall speciation in *Callithrix* given the historical geographic range separation of marmoset species described in Rylands *et al*. [17]. On the other hand, data from the PJ natural zone suggested a level of hybridization that does not disrupt the genetic integrity of species may be part of the evolutionary history of *Callithrix*. Thus, natural geographical barriers probably serve as important buffers against levels of hybridization that would erode the genetic integrity of separate marmoset lineages. Overall, we observed a lack of such gene flow barriers within forest fragments of the RJ anthropogenic zone, where we essentially observed complete collapse of genetic integrity within *C*. *penicillata* and *C*. *jacchus*. We note that the northern/southern autosomal population structure marmoset of the RJ anthropogenic hybrid zone suggests that BR-101 is a likely phyiscal barrier to gene flow across the zone. The physical isolation of marmoset populations among different forest fragments (as discussed above) also creates gene flow barriers within the RJ anthropogenic hybrid zone. However, when we look at the high level of admixture within individual sample locations on either side of the RJ hybrid zone (Fig 5 and Fig 6), our autosomal data indicate that individuals sampled within the RJ anthropogenic hybrid zone represent a hybrid swarm where parental genomes have been replaced with highly admixed hybrid genomes. From an evolutionary point of view, pure *C*. *jacchus* and *C*. *penicillata* genomes are probably extinct within the forest fragments of the anthropogenic RJ hybrid zone.

Other areas where *C*. *jacchus* and *C*. *penicillata* have been introduced outside of their natural distributions occur in the state of Rio de Janeiro, as well as in the Brazilian states of São Paulo and Minas Gerais (personal observation, IOS and VB). These areas include ranges of the three previously mentioned threatened *Callithrix* species. *Callithrix jacchus* and *C*. *penicillata* may pose an ecological as well as genetic threat to other marmoset species, since they are more morphologically specialized than other marmosets to exploit disturbed habitats [60], which characterize much of the Brazilian Atlantic Forest [61]. In the RJ anthropogenic zone, hybrid marmosets also may pose a threat to the endangered golden lion tamarin (*Leontopithecus rosalia*) [21]. These lion tamarins are part of the native biota of the RJ hybrid zone and share a similar biology and ecology to the introduced marmosets. Evidence from Ruiz-Miranada *et al*. [22] indicates that golden lion tamarins and exotic marmosets compete for similar resources.

Our data showed that hybridization is likely a natural part of the evolution of marmosets, yet it can also threaten the genetic integrity of marmoset species under human-induced conditions. As recommended by Allendorf *et al*. [10], the distinction between natural and anthropogenic hybridization should be made whenever possible in conservation decisions regarding marmosets. Additionally, demographic factors need to be taken into account regarding admixed populations, as marmoset species and populations can vary between combinations of invasive/native and threatened/stable. We recommend expanding genetic research on marmoset hybridization within both anthropogenic and natural contexts because such comparative data will be valuable in establishing the evolutionary and conservation values of pure and admixed populations. These data will become particularly indispensable if reports of recent marmoset hybridization continue to rise, increasing our need to understand the effects and to assess the value of admixed populations evolving under variable contexts and conditions.

## Supporting Information

S1 Data Genepop formatted file of raw microsatellite multilocus genotypes used in this study. (TXT)

S1 Fig File containing STRUCTURE *q*-value confidence intervals. (DOCX)

S1 Table Locations and latitude/longitude coordinates for captive and wild samples. (DOCX)

S2 Table PCR multiplexes of microsatellite loci. (DOCX)

S3 Table Locus-by-locus summary of various genetic diversity indices for *C*. *penicillata*. (DOCX)

S4 Table Locus-by-locus summary of various genetic diversity indices for *C*. *jacchus*. (DOCX)

S5 Table Locus-by-locus summary of various genetic diversity indices for the PJ natural hybrid zone. (DOCX)

S6 Table Locus-by-locus summary of various genetic diversity indices for the RJ anthropogenic hybrid zone. (DOCX)

S7 Table Allele frequencies uncorrected and corrected for presence of null alleles as observed within parental species and hybrid zones at each locus. (DOCX)

S8 Table STRUCTURE results for 10 different simulated data sets. (DOCX)

S9 Table BAPS results for 5 different simulated data sets. (DOCX)
