# Supplementary material for: Natural and Anthropogenic Hybridization in Two Species of Eastern Brazilian Marmosets (Callithrix jacchus and C. penicillata)
Source: PLoS One. 2015 Jun 10;10(6):e0127268. doi: 10.1371/journal.pone.0127268 (PMC4464756; doi:10.1371/journal.pone.0127268)
Supplement: S1 Fig — (DOCX) [file pone.0127268.s002.docx]

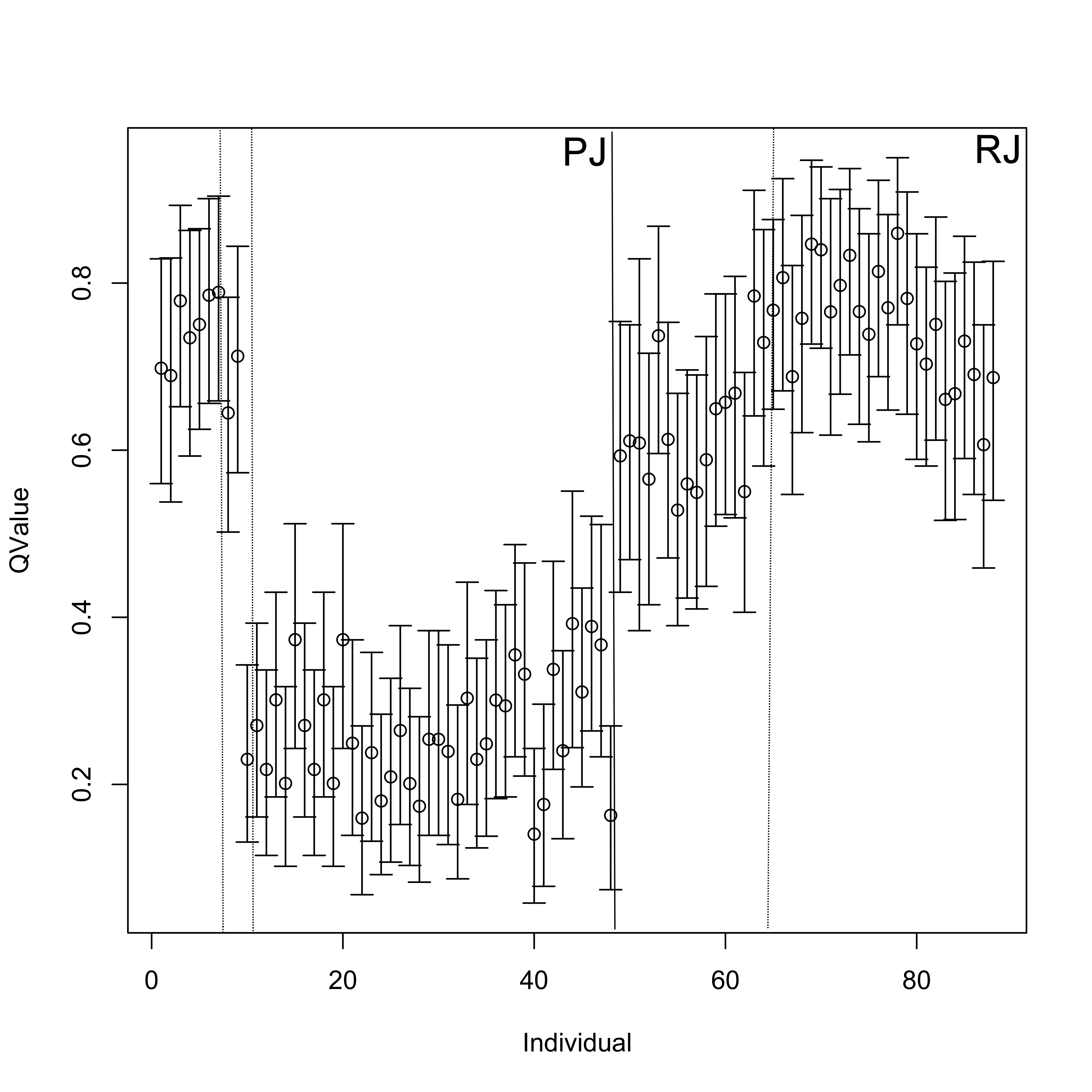


S1 Fig. Plot of STRUCTURE *q*-values and 90% confidence intervals for individuals sampled in the RJ and PJ hybrid zones. *Q*-values are in reference to *C. penicillata* (0 indicates no *C. penicillata* ancestry and 1 indicates full *C. penicillata* ancestry). A solid black line separates the PJ and RJ zones in the first and second respective halves of the plot. Dotted black lines within each hybrid zone plot separate the southern and northern portions of each zone. Also, in panel A, CEMAFAUNA captive marmosets are found between the two dotted black lines.
