# Supplementary material for: Natural and Anthropogenic Hybridization in Two Species of Eastern Brazilian Marmosets (Callithrix jacchus and C. penicillata)
Source: PLoS One. 2015 Jun 10;10(6):e0127268. doi: 10.1371/journal.pone.0127268 (PMC4464756; doi:10.1371/journal.pone.0127268)
Supplement: S1 Table — (DOCX) [file pone.0127268.s003.docx]

S1 Table. Locations and latitude/longitude coordinates for captive and wild samples.

| **Taxa** | **Sampling Location** | **Latitude/Longitude Coordinates** | **Samples** |
| --- | --- | --- | --- |
| RJ Hybrids | Boa Esperança | 22°39'5.40"S 42°26'26.92"W, 22°39'19.98"S 42°26'24.16"W | RJ024, RJ028-RJ032 |
|  | Fazenda Afetiva | 22°37'57.32"S 42°28'1.67"W, 22°37'56.52"S 42°28'3.69"W | RJ005-RJ013 |
|  | Fazenda dos Tamarins | 22°36'6.33"S 42°23'34.21"W | RJ016-RJ023 |
|  | House | 22°41'2.81"S 42°29'7.39"W | RJ001-RJ004 |
|  | Pesque Pague | 22°36'22.73"S 42°23'47.13"W, 22°36'22.28"S 42°23'47.75"W | RJ014,RJ015, RJ033, RJ034, RJ044-RJ046 |
|  | Ponto do Camarão | 22°36'23.09"S 42°24'12.47"W | RJ025-RJ027 |
|  | Rio Vermelho I | 22°42'58.02"S 42°33'52.27"W | RJ035-RJ037 |
|  | Rio Vermelho II | 22°43'18.36"S 42°34'50.64"W, 22°43'15.12"S 42°35'4.02"W | RJ038-RJ043 |
|  |  |  |  |
| PJ Hybrids | CEMAFAUNA^e^ | N/A | PJ035-PJ037 |
|  | Chácara Bom Jesus | 9°26'52.53"S 40°33'29.25"W | PJ064-PJ070 |
|  | Chácara do Senhor dos Santos | 9°24'33.76"S 40°30'47.06"W | PJ033 |
|  | Chácara Galo da Briga | 9°20'47.85"S 40°25'18.30"W | PJ058-PJ063 |
|  | Recanto do Sossego | 9°27'58.52"S 40°33'31.95"W | PJ030-PJ032 |
|  | Rio Verde | 9°14'31.59"S 40°18'39.96"W | PJ038-PJ042 |
|  | Sítio Caranaíba | 9°16'12.97"S 40°22'59.73"W | PJ050, PJ052-PJ056 |
|  | Sítio Picos | 9°15'50.69"S 40°19'19.24"W | PJ048, PJ049, PJ051, PJ057 |
|  | Sítio Porto da Cruz | 9°13'5.72"S 40°18'13.74"W | PJ043-PJ047 |
|  | Universidade do Estado da Bahia | 9°25'16.63"S 40°28'58.73"W | PJ028, PJ029,PJ034 |
|  |  |  |  |
| *C. penicillata* | Brasília Zoo, Brasília, DF | 15°50'40.12"S 47°56'35.83"W | CPE016, CPE017 |
|  | Callitrichid Research Center ^a^ | N/A | CPE001-CPE008 |
|  | Clube do Congresso, Brasília, DF | 15°46'12.90"S 47°49'59.83"W | CPE012, CPE013 |
|  | Condomínio Ouro Vermelho, Brasília, DF | 15°52'28.65"S 47°46'14.94"W | CPE027, CPE028 |
|  | CONTAG, Brasília, DF | 15°51'56.46"S 47°58'13.94"W | CPE020-CPE022 |
|  | Horto Florestal, MG | 21° 7' 15.60"S  42° 22' 2.50"W | CPE009-CPE011 |
|  | IBAMA CETAS, Goiânia, Goiás^b^ | N/A | CPE039-CPE043 |
|  | IBAMA CETAS, Recife, PB^b^ | N/A | CPE044-CPE046 |
|  | Instituto Israel Pinheiro, Brasília, DF | 15°48'11.03"S 47°47'57.49"W | CPE014, CPE015 |
|  | Jardim Botânico, Brasília, DF | 15°51'40.15"S 47°49'43.47"W | CPE029-CPE032, CPE035 |
|  | Parque dos Buritis, Goiânia, Goiás | 16°40'55.22"S 49°15'43.08"W | CPE038 |
|  | Q19 Conjunto 6, Brasília, DF | 15°45'1.15"S 47°50'34.10"W | CPE018, CPE019 |
|  | São Sebastião, Rua do Bosque, Brasília, DF | 15°54'31.51"S 47°45'26.26"W | CPE024 |
|  | School Mistress House, Goiânia, Goiás | 16°40'47.69"S 49°12'28.98"W | CPE036, CPE037 |
|  | SWPW Quadra 15, Conjunto 5, Brasília, DF | 15°54'38.07"S 47°57'10.64"W | CPE025, CPE026 |
|  | Vila Weslyn, Brasília, DF | 15°42'33.45"S 47°54'44.80"W | CPE023 |
|  |  |  |  |
| *C. jacchus* | Callitrichide Research Center ^a^ | N/A | CJA013, CJA014 |
|  | IBAMA CETAS, Recife, PE^b^ | N/A | CJA018-CJA041 |
|  | NEPRC^c^ | N/A | CJA002-CJA011 |
|  | Parque Dois Irmãos, Recife, PE, Tapacurá Reserve, PE^d^ | N/A | CJA043-CJA085 |

^a^Callitrichid Research Center, University of Nebraska at Omaha

^b^Wild Animal Triage Center, Brazilian Institute of the Environment and Natural Resources

^c^New England Primate Research Center

^d^Collected by Dr. Maria Adélia Borstelmann de Oliveira

^e^Center for Management of Fauna of the Caatinga
