# Supplementary material for: Natural and Anthropogenic Hybridization in Two Species of Eastern Brazilian Marmosets (Callithrix jacchus and C. penicillata)
Source: PLoS One. 2015 Jun 10;10(6):e0127268. doi: 10.1371/journal.pone.0127268 (PMC4464756; doi:10.1371/journal.pone.0127268)
Supplement: S2 Table — (DOCX) [file pone.0127268.s004.docx]

S2 Table. PCR multiplexes of microsatellite loci. Loci labeled as “excluded” were not included in a multiplex due to poor PCR amplification.

| **Multiplex Set** | **Locus Name** | **Fluorescent Dye** | **Size Range** | **Reference** |
| --- | --- | --- | --- | --- |
| 1 | caja1 | 6-FAM | 363-391 | Raveendran et al. (2008) |
| 1 | caja5 | NED | 233-250 | Raveendran et al. (2008) |
| 1 | caja9 | NED | 165-200 | Raveendran et al. (2008) |
| 1 | cj11 | 6-FAM | 114-130 | Nievergelt et al. (1998) |
| 2 | caja10 | VIC | 171-223 | Raveendran et al. (2008) |
| 2 | caja11 | PET | 234-258 | Raveendran et al. (2008) |
| 2 | caja13 | 6-FAM | 342-370 | Raveendran et al. (2008) |
| 3 | caja12 | NED | 212-266 | Raveendran et al. (2008) |
| 3 | caja16 | 6-FAM | 380-406 | Raveendran et al. (2008) |
| 3 | caja18 | PET | 297-315 | Raveendran et al. (2008) |
| 3 | cj14 | 6-FAM | 121-180 | Nievergelt et al. (1998) |
| 4 | cj13 | VIC | excluded | Nievergelt et al. (1998) |
| 4 | ham60 | VIC | 120-146 | Katoh et al. (2009) |
| 4 | ham96 | 6-FAM | 326-372 | Katoh et al. (2009) |
| 4 | ham181 | NED | 194-228 | Katoh et al. (2009) |
| 5 | caja14 | 6-FAM | 198-230 | Raveendran et al. (2008) |
| 5 | caja15 | VIC | 118-150 | Raveendran et al. (2008) |
| 5 | caja17 | 6-FAM | 352-426 | Raveendran et al. (2008) |
| 6 | caja19 | 6-FAM | 320-378 | Raveendran et al. (2008) |
| 6 | ham41 | 6-FAM | excluded | Katoh et al. (2009) |
| 6 | ham141 | VIC | 287-242 | Katoh et al. (2009) |
| 6 | ham55 | PET | 243-304 | Katoh et al. (2009) |
| 7 | cj6 | 6-FAM | 127-157 | Nievergelt et al. (1998) |
| 7 | ham30 | 6-FAM | 284-309 | Katoh et al. (2009) |
| 7 | ham1 | PET | 175-201 | Katoh et al. (2009) |
| 8 | ham3 | 6-FAM | 74-111 | Katoh et al. (2009) |
| 8 | ham6 | NED | excluded | Katoh et al. (2009) |
| 8 | ham57 | PET | 230-244 | Katoh et al. (2009) |
| 8 | lchu06 | VIC | 170-195 | Galbusera and Gillemot (2008) |
| 9 | ham100 | 6-FAM | 220-248 | Katoh et al. (2009) |
| 9 | ham116 | VIC | 273-298 | Katoh et al. (2009) |
| 9 | ham146 | 6-FAM | 128-153 | Katoh et al. (2009) |
| 10 | cj1 | NED | 117-177 | Nievergelt et al. (1998) |
| 10 | ham47 | PET | 278-302 | Katoh et al. (2009) |
| 10 | ham120 | VIC | 183-221 | Katoh et al. (2009) |
| 11 | ham26 | VIC | 163-186 | Katoh et al. (2009) |
| 11 | ham38 | 6-FAM | 257-285 | Katoh et al. (2009) |
| 12 | ham91 | 6-FAM | 128-160 | Katoh et al. (2009) |
| 12 | ham101 | VIC | 262-284 | Katoh et al. (2009) |
| 13 | ham107 | PET | 262-289 | Katoh et al. (2009) |
| 13 | ham150 | 6-FAM | 154-170 | Katoh et al. (2009) |
| 14 | ham8 | PET | 270-293 | Katoh et al. (2009) |
| 14 | ham102 | 6-FAM | 162-184 | Katoh et al. (2009) |
| 14 | ham103 | 6-FAM | 89-129 | Katoh et al. (2009) |
| 15 | ham79 | 6-FAM | 123-141 | Katoh et al. (2009) |
| 15 | ham123 | NED | 149-173 | Katoh et al. (2009) |
| 15 | ham184 | VIC | 169-204 | Katoh et al. (2009) |
| N/A | caja6 | NED | excluded | Nievergelt et al. (1998) |
| N/A | cj7 | 6-FAM | excluded | Raveendran et al. (2008) |
| N/A | cj15 | VIC | excluded | Raveendran et al. (2008) |
