# Supplementary material for: Natural and Anthropogenic Hybridization in Two Species of Eastern Brazilian Marmosets (Callithrix jacchus and C. penicillata)
Source: PLoS One. 2015 Jun 10;10(6):e0127268. doi: 10.1371/journal.pone.0127268 (PMC4464756; doi:10.1371/journal.pone.0127268)
Supplement: S3 Table — (DOCX) [file pone.0127268.s005.docx]

S3 Table. Locus-by-locus summary of various genetic diversity indices for *C*. *penicillata.* N is number of individuals sampled at a locus, A is the number of alleles at a locus, R is allelic richness, r is EM null allele frequency, Ho is observed heterozygosity, H_E_ is expected heterozygosity, F_IS_ is the inbreeding coefficient. F_IS_ values in bold indicate loci which were flagged by Microchecker for the possible presence of null alleles. F_IS_ values that are starred are significant for Hardy-Weinberg disequilibrium for various *P-*values as follows: * = p<0.05, ** = p<0.01,*** = p<0.001.

| **Locus** | ***C. penicillata*** | | | | | | |
| --- | --- | --- | --- | --- | --- | --- | --- |
|  | **N** | ***A*** | **R** | **r** | **H_o_** | **H_E_** | **F_IS_** |
| caja1 | 41 | 11 | 10.516 | 0.000 | 0.829 | 0.833 | 0.004 |
| caja10 | 29 | 16 | 16.000 | 0.236 | 0.379 | 0.849 | **0.553***** |
| caja11 | 40 | 10 | 9.412 | 0.040 | 0.775 | 0.859 | 0.098 |
| caja12 | 37 | 16 | 14.641 | 0.134 | 0.459 | 0.714 | **0.356***** |
| caja13 | 41 | 10 | 9.159 | 0.053 | 0.561 | 0.702 | 0.2* |
| caja14 | 40 | 11 | 10.504 | 0.012 | 0.800 | 0.836 | 0.043 |
| caja15 | 41 | 10 | 9.246 | 0.032 | 0.732 | 0.805 | 0.092 |
| caja16 | 36 | 9 | 8.540 | 0.166 | 0.500 | 0.799 | **0.374***** |
| caja17 | 39 | 17 | 15.841 | 0.049 | 0.795 | 0.920 | 0.136 |
| caja18 | 37 | 8 | 7.947 | 0.007 | 0.811 | 0.803 | -0.009 |
| caja19 | 40 | 10 | 9.334 | 0.146 | 0.500 | 0.782 | **0.361***** |
| caja5 | 39 | 7 | 6.602 | 0.118 | 0.436 | 0.639 | 0.317 |
| caja9 | 36 | 8 | 7.792 | 0.138 | 0.528 | 0.761 | **0.307**** |
| cj1 | 39 | 15 | 14.325 | 0.061 | 0.769 | 0.914 | 0.158 |
| cj11 | 41 | 5 | 4.415 | 0.203 | 0.293 | 0.631 | **0.536***** |
| cj14 | 39 | 13 | 12.070 | 0.088 | 0.564 | 0.756 | **0.254*** |
| cj6 | 41 | 12 | 11.493 | 0.058 | 0.780 | 0.874 | 0.107 |
| ham1 | 39 | 10 | 9.442 | 0.068 | 0.718 | 0.843 | 0.149 |
| ham100 | 40 | 11 | 10.811 | 0.144 | 0.575 | 0.870 | **0.339***** |
| ham101 | 38 | 8 | 7.758 | 0.144 | 0.553 | 0.812 | **0.32***** |
| ham102 | 36 | 12 | 11.727 | 0.045 | 0.778 | 0.893 | 0.129 |
| ham103 | 35 | 11 | 10.282 | 0.154 | 0.486 | 0.781 | **0.378*** |
| Ham107 | 38 | 11 | 10.351 | 0.100 | 0.553 | 0.722 | **0.235***** |
| ham116 | 39 | 10 | 9.291 | 0.154 | 0.385 | 0.671 | **0.427***** |
| ham120 | 40 | 13 | 11.537 | 0.058 | 0.600 | 0.695 | 0.137 |
| ham123 | 29 | 6 | 6.000 | 0.161 | 0.517 | 0.828 | **0.375*** |
| ham141 | 32 | 15 | 14.595 | 0.226 | 0.438 | 0.876 | **0.5***** |
| ham146 | 39 | 9 | 8.345 | 0.001 | 0.590 | 0.586 | -0.006 |
| Ham150 | 40 | 7 | 6.780 | 0.000 | 0.725 | 0.744 | 0.026 |
| ham181 | 40 | 12 | 11.280 | 0.058 | 0.750 | 0.873 | 0.141 |
| ham184 | 35 | 15 | 14.425 | 0.014 | 0.857 | 0.891 | 0.038 |
| ham26 | 30 | 7 | 6.966 | 0.155 | 0.400 | 0.692 | **0.422**** |
| ham3 | 39 | 12 | 11.896 | 0.019 | 0.846 | 0.871 | 0.029 |
| ham30 | 41 | 9 | 7.933 | 0.078 | 0.561 | 0.733 | 0.235 |
| ham38 | 38 | 13 | 11.891 | 0.087 | 0.684 | 0.813 | 0.158 |
| ham47 | 40 | 9 | 8.424 | 0.071 | 0.675 | 0.771 | 0.125 |
| ham55 | 40 | 12 | 10.711 | 0.107 | 0.425 | 0.671 | **0.367***** |
| ham57 | 41 | 6 | 5.915 | 0.092 | 0.537 | 0.664 | 0.192 |
| ham60 | 39 | 8 | 7.423 | 0.162 | 0.462 | 0.741 | **0.377***** |
| ham79 | 35 | 9 | 8.945 | 0.068 | 0.686 | 0.768 | 0.107 |
| ham8 | 31 | 13 | 12.803 | 0.082 | 0.677 | 0.879 | **0.229*** |
| ham91 | 41 | 13 | 11.154 | 0.040 | 0.780 | 0.830 | 0.060 |
| ham96 | 34 | 13 | 12.622 | 0.034 | 0.794 | 0.854 | 0.07* |
| lchu06 | 40 | 16 | 14.982 | 0.106 | 0.700 | 0.920 | **0.239***** |
| Per Locus Average | 37.841 | 10.864 | 10.276 | 0.090 | 0.620 | 0.790 | 0.216 |
