# Supplementary material for: Natural and Anthropogenic Hybridization in Two Species of Eastern Brazilian Marmosets (Callithrix jacchus and C. penicillata)
Source: PLoS One. 2015 Jun 10;10(6):e0127268. doi: 10.1371/journal.pone.0127268 (PMC4464756; doi:10.1371/journal.pone.0127268)
Supplement: S4 Table — (DOCX) [file pone.0127268.s006.docx]

S4 Table. Locus-by-locus summary of various genetic diversity indices for *C. jacchus.* N is number of individuals sampled at a locus, A is the number of alleles at a locus, R is allelic richness, r is EM null allele frequency, Ho is observed heterozygosity, H_E_ is expected heterozygosity, F_IS_ is the inbreeding coefficient. F_IS_ values in bold indicate loci which were flagged by Microchecker for the possible presence of null alleles. F_IS_ values that are starred are significant for Hardy-Weinberg disequilibrium for various *P-*values as follows: * = p<0.05, ** = p<0.01,*** = p<0.001.

| **Locus** | ***C. jacchus*** | | | | | | |
| --- | --- | --- | --- | --- | --- | --- | --- |
|  | **N** | ***A*** | **R** | **r** | **H_o_** | **H_E_** | **F_IS_** |
| caja1 | 50 | 9 | 8.489 | 0.101 | 0.620 | 0.827 | 0.250 |
| caja10 | 59 | 12 | 9.803 | 0.004 | 0.814 | 0.848 | 0.041 |
| caja11 | 59 | 6 | 4.215 | 0.000 | 0.220 | 0.205 | -0.076 |
| caja12 | 54 | 11 | 8.754 | 0.022 | 0.741 | 0.757 | 0.022 |
| caja13 | 58 | 7 | 6.906 | 0.002 | 0.759 | 0.750 | -0.011 |
| caja14 | 60 | 7 | 6.210 | 0.076 | 0.500 | 0.645 | 0.225 |
| caja15 | 58 | 8 | 7.471 | 0.032 | 0.776 | 0.841 | 0.078 |
| caja16 | 52 | 6 | 5.875 | 0.081 | 0.423 | 0.568 | **0.256**** |
| caja17 | 60 | 11 | 9.164 | 0.002 | 0.583 | 0.620 | 0.059 |
| caja18 | 53 | 5 | 4.508 | 0.113 | 0.491 | 0.687 | 0.286 |
| caja19 | 52 | 10 | 8.032 | 0.067 | 0.596 | 0.735 | 0.189 |
| caja5 | 53 | 3 | 2.997 | 0.072 | 0.491 | 0.564 | 0.131 |
| caja9 | 55 | 7 | 6.378 | 0.005 | 0.673 | 0.721 | 0.067 |
| cj1 | 57 | 8 | 6.706 | 0.000 | 0.544 | 0.546 | 0.003 |
| cj11 | 48 | 3 | 2.978 | 0.029 | 0.542 | 0.518 | -0.046 |
| cj14 | 51 | 10 | 8.706 | 0.038 | 0.725 | 0.793 | 0.085 |
| cj6 | 59 | 8 | 7.060 | 0.068 | 0.424 | 0.544 | 0.221 |
| ham1 | 57 | 9 | 7.777 | 0.000 | 0.807 | 0.831 | 0.029 |
| ham100 | 57 | 8 | 7.643 | 0.000 | 0.825 | 0.842 | 0.020 |
| ham101 | 53 | 9 | 8.035 | 0.010 | 0.811 | 0.759 | -0.068 |
| ham102 | 55 | 5 | 4.731 | 0.056 | 0.582 | 0.624 | 0.068 |
| ham103 | 55 | 12 | 9.619 | 0.013 | 0.745 | 0.776 | 0.039 |
| Ham107 | 59 | 10 | 7.550 | 0.018 | 0.729 | 0.716 | -0.017 |
| ham116 | 50 | 6 | 5.581 | 0.241 | 0.200 | 0.552 | **0.637***** |
| ham120 | 56 | 10 | 7.830 | 0.071 | 0.339 | 0.431 | 0.213 |
| ham123 | 57 | 12 | 10.162 | 0.025 | 0.719 | 0.787 | 0.086 |
| ham141 | 54 | 10 | 8.260 | 0.000 | 0.870 | 0.800 | -0.087 |
| ham146 | 54 | 8 | 5.589 | 0.013 | 0.519 | 0.575 | 0.099 |
| Ham150 | 53 | 6 | 5.091 | 0.057 | 0.547 | 0.686 | 0.203 |
| ham181 | 54 | 11 | 9.111 | 0.000 | 0.759 | 0.800 | 0.050 |
| ham184 | 55 | 11 | 9.580 | 0.000 | 0.818 | 0.831 | 0.015 |
| ham26 | 57 | 9 | 7.797 | 0.013 | 0.789 | 0.733 | -0.078 |
| ham3 | 59 | 9 | 7.801 | 0.039 | 0.627 | 0.680 | 0.077** |
| ham30 | 56 | 9 | 8.197 | 0.030 | 0.661 | 0.736 | 0.103 |
| ham38 | 60 | 9 | 7.399 | 0.061 | 0.600 | 0.665 | 0.097 |
| ham47 | 54 | 9 | 7.758 | 0.003 | 0.611 | 0.615 | 0.007 |
| ham55 | 55 | 9 | 6.918 | 0.000 | 0.673 | 0.680 | 0.011 |
| ham57 | 59 | 5 | 4.424 | 0.036 | 0.441 | 0.527 | 0.165 |
| ham60 | 59 | 8 | 6.150 | 0.105 | 0.288 | 0.402 | 0.283 |
| ham79 | 51 | 7 | 6.936 | 0.065 | 0.686 | 0.786 | 0.127* |
| ham8 | 53 | 8 | 6.188 | 0.005 | 0.509 | 0.538 | 0.053 |
| ham91 | 61 | 11 | 8.396 | 0.029 | 0.656 | 0.695 | 0.056** |
| ham96 | 58 | 8 | 6.792 | 0.000 | 0.759 | 0.662 | -0.146 |
| lchu06 | 56 | 10 | 7.730 | 0.014 | 0.696 | 0.718 | 0.030 |
| Per Locus Average | 55.568 | 8.386 | 7.120 | 0.037 | 0.618 | 0.673 | 0.082 |
