# Supplementary material for: Natural and Anthropogenic Hybridization in Two Species of Eastern Brazilian Marmosets (Callithrix jacchus and C. penicillata)
Source: PLoS One. 2015 Jun 10;10(6):e0127268. doi: 10.1371/journal.pone.0127268 (PMC4464756; doi:10.1371/journal.pone.0127268)
Supplement: S5 Table — (DOCX) [file pone.0127268.s007.docx]

S5 Table. Locus-by-locus summary of various genetic diversity indices for the PJ natural hybrid zone. N is number of individuals sampled at a locus, A is the number of alleles at a locus, R is allelic richness, r is EM null allele frequency, Ho is observed heterozygosity, H_E_ is expected heterozygosity, F_IS_ is the inbreeding coefficient. F_IS_ values in bold indicate loci which were flagged by Microchecker for the possible presence of null alleles. F_IS_ values that are starred are significant for Hardy-Weinberg disequilibrium for various *P-*values as follows: * = p<0.05, ** = p<0.01,*** = p<0.001.

| **Locus** | **PJ Zone** | | | | | | |
| --- | --- | --- | --- | --- | --- | --- | --- |
|  | **N** | ***A*** | **R** | **r** | **H_o_** | **H_E_** | **F_IS_** |
| caja1 | 42 | 11 | 9.705 | 0.009 | 0.738 | 0.747 | 0.011 |
| caja10 | 42 | 12 | 11.598 | 0.042 | 0.810 | 0.889 | 0.089*** |
| caja11 | 42 | 9 | 8.256 | 0.000 | 0.786 | 0.747 | -0.052 |
| caja12 | 42 | 11 | 9.587 | 0.005 | 0.548 | 0.610 | 0.103 |
| caja13 | 42 | 7 | 6.683 | 0.021 | 0.714 | 0.731 | 0.022 |
| caja14 | 42 | 10 | 8.970 | 0.068 | 0.643 | 0.788 | **0.184*** |
| caja15 | 43 | 8 | 7.467 | 0.135 | 0.442 | 0.687 | **0.357***** |
| caja16 | 37 | 7 | 6.686 | 0.013 | 0.622 | 0.592 | -0.050 |
| caja17 | 42 | 10 | 9.194 | 0.061 | 0.714 | 0.845 | 0.155 |
| caja18 | 41 | 8 | 7.383 | 0.069 | 0.415 | 0.577 | **0.282*** |
| caja19 | 37 | 4 | 3.784 | 0.228 | 0.189 | 0.519 | **0.635***** |
| caja5 | 42 | 5 | 4.682 | 0.131 | 0.429 | 0.612 | **0.3**** |
| caja9 | 41 | 9 | 8.241 | 0.036 | 0.659 | 0.775 | 0.150 |
| cj1 | 41 | 8 | 7.097 | 0.149 | 0.390 | 0.653 | **0.403*** |
| cj11 | 40 | 4 | 3.720 | 0.135 | 0.275 | 0.471 | 0.416 |
| cj14 | 40 | 13 | 11.421 | 0.026 | 0.725 | 0.742 | 0.022 |
| cj6 | 40 | 5 | 4.889 | 0.050 | 0.425 | 0.529 | 0.197 |
| ham1 | 40 | 10 | 9.284 | 0.035 | 0.675 | 0.754 | 0.105 |
| ham100 | 37 | 9 | 8.522 | 0.036 | 0.757 | 0.800 | 0.054 |
| ham101 | 41 | 8 | 7.518 | 0.040 | 0.659 | 0.764 | 0.138 |
| ham102 | 41 | 8 | 7.677 | 0.028 | 0.756 | 0.804 | 0.059 |
| ham103 | 29 | 9 | 9.000 | 0.288 | 0.276 | 0.797 | **0.654***** |
| Ham107 | 40 | 9 | 8.349 | 0.097 | 0.525 | 0.677 | 0.224 |
| ham116 | 35 | 6 | 5.827 | 0.260 | 0.171 | 0.609 | **0.718***** |
| ham120 | 40 | 9 | 8.165 | 0.000 | 0.675 | 0.662 | -0.019 |
| ham123 | 38 | 10 | 9.641 | 0.053 | 0.737 | 0.808 | 0.088 |
| ham141 | 32 | 8 | 7.898 | 0.094 | 0.625 | 0.791 | 0.210 |
| ham146 | 39 | 6 | 5.472 | 0.024 | 0.410 | 0.464 | 0.115 |
| Ham150 | 38 | 6 | 5.986 | 0.106 | 0.526 | 0.692 | 0.240 |
| ham181 | 40 | 12 | 10.514 | 0.069 | 0.625 | 0.771 | 0.189 |
| ham184 | 36 | 6 | 5.929 | 0.122 | 0.556 | 0.753 | 0.262 |
| ham26 | 42 | 9 | 8.167 | 0.005 | 0.786 | 0.768 | -0.023 |
| ham3 | 38 | 9 | 8.459 | 0.052 | 0.684 | 0.787 | 0.131 |
| ham30 | 40 | 8 | 7.614 | 0.004 | 0.875 | 0.794 | -0.102 |
| ham38 | 39 | 11 | 10.146 | 0.118 | 0.564 | 0.791 | **0.287*** |
| ham47 | 41 | 7 | 6.970 | 0.011 | 0.780 | 0.789 | 0.011 |
| ham55 | 36 | 7 | 6.576 | 0.058 | 0.556 | 0.656 | 0.154 |
| ham57 | 41 | 5 | 4.701 | 0.071 | 0.488 | 0.592 | 0.176 |
| ham60 | 40 | 7 | 6.448 | 0.116 | 0.500 | 0.696 | 0.281 |
| ham79 | 35 | 4 | 3.829 | 0.043 | 0.543 | 0.636 | 0.146 |
| ham8 | 39 | 11 | 9.847 | 0.049 | 0.590 | 0.696 | 0.152 |
| ham91 | 43 | 11 | 9.448 | 0.023 | 0.488 | 0.575 | 0.151* |
| ham96 | 36 | 9 | 8.345 | 0.009 | 0.750 | 0.767 | 0.022 |
| lchu06 | 41 | 10 | 9.433 | 0.140 | 0.463 | 0.710 | **0.347***** |
| Per Locus Average | 39.386 | 8.295 | 7.707 | 0.071 | 0.581 | 0.703 | 0.173 |
