# Supplementary material for: Natural and Anthropogenic Hybridization in Two Species of Eastern Brazilian Marmosets (Callithrix jacchus and C. penicillata)
Source: PLoS One. 2015 Jun 10;10(6):e0127268. doi: 10.1371/journal.pone.0127268 (PMC4464756; doi:10.1371/journal.pone.0127268)
Supplement: S6 Table — (DOCX) [file pone.0127268.s008.docx]

S6 Table. Locus-by-locus summary of various genetic diversity indices for the RJ anthropogenic hybrid zone. N is number of individuals sampled at a locus, A is the number of alleles at a locus, R is allelic richness, r is EM null allele frequency, Ho is observed heterozygosity, H_E_ is expected heterozygosity, F_IS_ is the inbreeding coefficient. F_IS_ values in bold indicate loci which were flagged by Microchecker for the possible presence of null alleles. F_IS_ values that are starred are significant for Hardy-Weinberg disequilibrium for various *P-*values as follows: * = p<0.05, ** = p<0.01,*** = p<0.001.

| **Locus** | **RJ Zone** | | | | | | |
| --- | --- | --- | --- | --- | --- | --- | --- |
|  | **N** | ***A*** | **R** | **r** | **H_o_** | **H_E_** | **F_IS_** |
| caja1 | 43 | 9 | 8.004 | 0.000 | 0.581 | 0.666 | 0.126 |
| caja10 | 35 | 7 | 6.991 | 0.165 | 0.486 | 0.808 | **0.399***** |
| caja11 | 44 | 6 | 5.995 | 0.024 | 0.727 | 0.786 | 0.075* |
| caja12 | 43 | 9 | 8.235 | 0.029 | 0.721 | 0.803 | 0.102 |
| caja13 | 44 | 6 | 5.432 | 0.000 | 0.705 | 0.667 | -0.057 |
| caja14 | 43 | 8 | 7.330 | 0.000 | 0.791 | 0.751 | -0.052 |
| caja15 | 40 | 6 | 5.652 | 0.119 | 0.500 | 0.710 | **0.295***** |
| caja16 | 37 | 5 | 4.784 | 0.092 | 0.541 | 0.694 | 0.222 |
| caja17 | 44 | 8 | 7.984 | 0.011 | 0.750 | 0.812 | 0.076 |
| caja18 | 45 | 6 | 5.520 | 0.072 | 0.556 | 0.674 | 0.176 |
| caja19 | 40 | 5 | 4.718 | 0.083 | 0.250 | 0.335 | 0.253 |
| caja5 | 42 | 4 | 4.000 | 0.000 | 0.738 | 0.700 | -0.055 |
| caja9 | 42 | 3 | 2.690 | 0.121 | 0.214 | 0.361 | 0.407 |
| cj1 | 43 | 5 | 4.865 | 0.020 | 0.628 | 0.681 | 0.078 |
| cj11 | 41 | 3 | 2.998 | 0.000 | 0.415 | 0.396 | -0.048 |
| cj14 | 45 | 10 | 8.886 | 0.014 | 0.822 | 0.839 | 0.020 |
| cj6 | 43 | 8 | 7.465 | 0.068 | 0.488 | 0.671 | 0.272 |
| ham1 | 41 | 7 | 6.828 | 0.019 | 0.634 | 0.745 | 0.149 |
| ham100 | 43 | 6 | 5.894 | 0.059 | 0.605 | 0.707 | 0.145 |
| ham101 | 43 | 7 | 6.571 | 0.169 | 0.465 | 0.771 | **0.397***** |
| ham102 | 36 | 7 | 6.734 | 0.030 | 0.694 | 0.779 | 0.109 |
| ham103 | 34 | 6 | 6.000 | 0.165 | 0.441 | 0.706 | 0.375 |
| Ham107 | 43 | 7 | 6.561 | 0.109 | 0.581 | 0.769 | 0.244 |
| ham116 | 42 | 7 | 6.879 | 0.001 | 0.786 | 0.805 | 0.025 |
| ham120 | 43 | 6 | 5.571 | 0.000 | 0.744 | 0.742 | -0.003 |
| ham123 | 39 | 5 | 4.740 | 0.107 | 0.436 | 0.656 | **0.335***** |
| ham141 | 40 | 9 | 8.633 | 0.000 | 0.850 | 0.831 | -0.022 |
| ham146 | 43 | 6 | 5.245 | 0.013 | 0.605 | 0.578 | -0.047 |
| Ham150 | 42 | 7 | 6.992 | 0.000 | 0.786 | 0.832 | 0.055 |
| ham181 | 42 | 8 | 7.663 | 0.000 | 0.857 | 0.838 | -0.023 |
| ham184 | 36 | 7 | 6.769 | 0.007 | 0.722 | 0.716 | -0.009 |
| ham26 | 43 | 8 | 7.236 | 0.053 | 0.698 | 0.797 | 0.124 |
| ham3 | 42 | 8 | 7.655 | 0.012 | 0.714 | 0.769 | 0.071 |
| ham30 | 43 | 6 | 5.665 | 0.050 | 0.674 | 0.766 | 0.120 |
| ham38 | 42 | 10 | 9.346 | 0.085 | 0.690 | 0.870 | 0.206 |
| ham47 | 43 | 7 | 6.761 | 0.035 | 0.698 | 0.779 | 0.104 |
| ham55 | 37 | 8 | 7.730 | 0.000 | 0.595 | 0.700 | 0.151 |
| ham57 | 44 | 6 | 5.963 | 0.007 | 0.750 | 0.780 | 0.039 |
| ham60 | 42 | 6 | 5.972 | 0.045 | 0.643 | 0.764 | 0.158 |
| ham79 | 39 | 7 | 6.680 | 0.121 | 0.487 | 0.727 | **0.33**** |
| ham8 | 36 | 9 | 8.575 | 0.023 | 0.778 | 0.835 | 0.069 |
| ham91 | 43 | 6 | 5.990 | 0.018 | 0.744 | 0.806 | 0.077 |
| ham96 | 40 | 6 | 5.702 | 0.038 | 0.550 | 0.649 | 0.152 |
| lchu06 | 42 | 7 | 6.879 | 0.036 | 0.738 | 0.802 | 0.080 |
| Per Locus Average | 41.295 | 6.750 | 6.427 | 0.046 | 0.634 | 0.724 | 0.125 |
