# Supplementary material for: Natural and Anthropogenic Hybridization in Two Species of Eastern Brazilian Marmosets (Callithrix jacchus and C. penicillata)
Source: PLoS One. 2015 Jun 10;10(6):e0127268. doi: 10.1371/journal.pone.0127268 (PMC4464756; doi:10.1371/journal.pone.0127268)
Supplement: S7 Table — (DOCX) [file pone.0127268.s009.docx]

S7 Table. Allele frequencies uncorrected and corrected for presence of null alleles as observed within parental species and hybrid zones at each locus. L indicates locus column, R indicates column of uncorrected raw frequencies, and C indicates column of corrected frequencies. Cells labeled as “NULL” indicate remaining amount of corrected allele frequencies that represents unobserved null alleles. Absence of an allele within a particular species or hybrid zone is indicated by a “-” symbol.

| **Locus** | **Allele** | ***C. penicillata*** | | ***C. jacchus*** | | **PJ Zone** | | **RJ Zone** | |
| --- | --- | --- | --- | --- | --- | --- | --- | --- | --- |
|  |  | **R** | **C** | **R** | **C** | **R** | **C** | **R** | **C** |
| **caja1** | **363** | 0.012 | 0.012 | - | - | 0.012 | 0.012 | - | - |
|  | **365** | 0.061 | 0.061 | - | - | 0.012 | 0.012 | 0.012 | 0.012 |
|  | **367** | 0.073 | 0.073 | - | - | 0.036 | 0.036 | 0.547 | 0.546 |
|  | **368** | - | - | - | - | - | - | 0.012 | 0.012 |
|  | **369** | - | - | - | - | 0.071 | 0.069 | - | - |
|  | **371** | 0.024 | 0.024 | - | - | - | - | 0.047 | 0.047 |
|  | **373** | 0.341 | 0.341 | 0.030 | 0.021 | 0.060 | 0.060 | 0.012 | 0.012 |
|  | **375** | 0.146 | 0.146 | 0.060 | 0.060 | 0.036 | 0.036 | 0.047 | 0.047 |
|  | **377** | 0.122 | 0.122 | 0.090 | 0.083 | - | - | - | - |
|  | **379** | 0.073 | 0.073 | 0.230 | 0.194 | 0.179 | 0.175 | - | - |
|  | **381** | 0.085 | 0.085 | 0.260 | 0.237 | 0.452 | 0.449 | 0.081 | 0.081 |
|  | **382** | - | - | - | - | 0.012 | 0.012 | - | - |
|  | **383** | 0.037 | 0.037 | 0.060 | 0.060 | 0.119 | 0.119 | 0.163 | 0.163 |
|  | **385** | 0.024 | 0.024 | 0.210 | 0.184 | - | - | 0.081 | 0.081 |
|  | **387** | - | - | 0.050 | 0.050 | - | - | - | - |
|  | **389** | - | - | 0.010 | 0.010 | - | - | - | - |
|  | **391** | - | - | - | - | 0.012 | 0.012 | - | - |
|  | **NULL** | NA | - | NA | 0.101 | NA | 0.009 | NA | - |
| **caja5** | **233** | 0.038 | 0.038 | - | - | - | - | - | - |
|  | **235** | - | - | - | - | 0.012 | 0.012 | - | - |
|  | **239** | 0.013 | 0.013 | 0.443 | 0.417 | 0.357 | 0.314 | 0.417 | 0.417 |
|  | **241** | 0.295 | 0.258 | 0.066 | 0.052 | 0.512 | 0.456 | 0.286 | 0.286 |
|  | **243** | 0.526 | 0.465 | 0.491 | 0.459 | 0.071 | 0.041 | 0.083 | 0.083 |
|  | **245** | 0.077 | 0.056 | - | - | 0.048 | 0.048 | 0.214 | 0.214 |
|  | **247** | 0.026 | 0.026 | - | - | - | - | - | - |
|  | **250** | 0.026 | 0.026 | - | - | - | - | - | - |
|  | **NULL** | NA | 0.118 | NA | 0.072 | NA | 0.131 | NA | - |
| **caja9** | **165** | 0.042 | 0.029 | - | - | - | - | - | - |
|  | **168** | 0.333 | 0.278 | - | - | - |  | - | - |
|  | **169** | 0.056 | 0.031 | - | - | - | - | - | - |
|  | **178** | 0.097 | 0.097 | - | - | 0.024 | 0.024 | - | - |
|  | **180** | 0.014 | 0.014 | 0.027 | 0.027 | 0.012 | 0.012 | 0.012 | 0.012 |
|  | **182** | 0.347 | 0.315 | 0.455 | 0.452 | 0.268 | 0.012 | 0.774 | 0.687 |
|  | **184** | 0.069 | 0.069 | 0.173 | 0.172 | 0.220 | 0.252 | - | - |
|  | **186** | 0.042 | 0.029 | - | - | 0.012 | 0.213 | - | - |
|  | **187** | - | - | 0.036 | 0.036 | - |  | - | - |
|  | **189** | - | - | - | - | 0.061 | 0.061 | - | - |
|  | **191** | - | - | 0.009 | 0.009 | 0.024 | 0.024 | - | - |
|  | **193** | - | - | 0.182 | 0.180 | 0.329 | 0.316 | 0.214 | 0.180 |
|  | **196** | - | - | 0.118 | 0.118 | - |  | - | - |
|  | **200** | - | - | - | - | 0.049 | 0.049 | - | - |
|  | **NULL** | NA | 0.138 | NA | 0.005 | NA | 0.036 | NA | 0.121 |
| **caja10** | **171** | - | - | 0.102 | 0.101 | - | - | - | - |
|  | **179** | 0.017 | 0.017 | 0.008 | 0.008 | 0.060 | 0.052 | - | - |
|  | **181** | 0.052 | 0.036 | 0.008 | 0.008 | 0.036 | 0.036 | - | - |
|  | **183** | - | - | 0.042 | 0.042 | 0.036 | 0.036 | - | - |
|  | **185** | 0.034 | 0.034 | 0.203 | 0.202 | 0.036 | 0.036 | 0.257 | 0.214 |
|  | **187** | 0.052 | 0.052 | 0.178 | 0.177 | 0.179 | 0.171 | - | - |
|  | **189** | 0.017 | 0.017 | 0.237 | 0.237 | 0.202 | 0.199 | - | - |
|  | **191** | 0.034 | 0.034 | 0.110 | 0.110 | 0.119 | 0.119 | - | - |
|  | **193** | - | - | 0.068 | 0.068 | 0.048 | 0.048 | 0.286 | 0.226 |
|  | **195** | - | - | - | - | 0.071 | 0.071 | 0.043 | 0.043 |
|  | **196** | 0.034 | 0.018 | 0.008 | 0.008 | - | - | - | - |
|  | **197** | - | - | 0.008 | 0.008 | - | - | - | - |
|  | **198** | 0.362 | 0.249 | - | - | - | - | - | - |
|  | **200** | 0.086 | 0.055 | 0.025 | 0.025 | - | - | - | - |
|  | **201** | - | - | - | - | 0.119 | 0.109 | - | - |
|  | **203** | 0.069 | 0.037 | - | - | - | - | 0.200 | 0.162 |
|  | **205** | 0.034 | 0.034 | - | - | - | - | 0.114 | 0.103 |
|  | **207** | 0.138 | 0.110 | - | - | - | - | 0.043 | 0.030 |
|  | **209** | 0.017 | 0.017 | - | - | 0.012 | 0.012 | - | - |
|  | **211** | 0.017 | 0.017 | - | - | 0.083 | 0.070 | - | - |
|  | **213** | 0.017 | 0.017 | - | - | - | - | - | - |
|  | **215** | - | - | - | - | - | - | 0.057 | 0.057 |
|  | **223** | 0.017 | 0.017 | - | - | - | - | - | - |
|  | **NULL** | NA | 0.236 | NA | 0.004 | NA | 0.042 | NA | 0.165 |
| **caja11** | **234** | - | - | 0.008 | 0.008 | 0.036 | 0.036 | - | - |
|  | **236** | - | - | 0.008 | 0.008 | 0.012 | 0.012 | - | - |
|  | **238** | 0.163 | 0.158 | 0.890 | 0.890 | 0.381 | 0.381 | 0.170 | 0.170 |
|  | **239** | - | - | - | - | 0.024 | 0.024 | - | - |
|  | **240** | - | - | 0.068 | 0.068 | - | - | 0.193 | 0.184 |
|  | **242** | 0.013 | 0.013 | - | - | - | - | - | - |
|  | **244** | 0.125 | 0.115 | 0.017 | 0.017 | 0.060 | 0.060 | - | - |
|  | **246** | 0.037 | 0.038 | - | - | 0.107 | 0.107 | 0.159 | 0.159 |
|  | **248** | 0.150 | 0.146 | 0.008 | 0.008 | 0.310 | 0.310 | 0.352 | 0.342 |
|  | **250** | 0.113 | 0.113 | - | - | 0.060 | 0.060 | 0.068 | 0.063 |
|  | **251** | - | - | - | - | 0.012 | 0.012 | - | - |
|  | **252** | 0.250 | 0.241 | - | - | - | - | 0.057 | 0.057 |
|  | **254** | 0.037 | 0.038 | - | - | - | - | - | - |
|  | **256** | 0.100 | 0.088 | - | - | - | - | - | - |
|  | **258** | 0.013 | 0.013 | - | - | - | - | - | - |
|  | **NULL** | NA | 0.040 | NA | - | NA | - | NA | 0.024 |
| **caja12** | **212** | - | - | 0.009 | 0.009 | - | - | - | - |
|  | **219** | 0.054 | 0.042 | - | - | 0.036 | 0.036 | - | - |
|  | **221** | - | - | 0.324 | 0.318 | 0.131 | 0.129 | - | - |
|  | **223** | - | - | - | - | - | - | 0.291 | 0.281 |
|  | **225** | 0.014 | 0.014 | 0.009 | 0.009 | 0.012 | 0.012 | - | - |
|  | **227** | - | - | 0.028 | 0.022 | - | - | 0.070 | 0.070 |
|  | **228** | - | - | - | - | - | - | 0.012 | 0.012 |
|  | **229** | 0.027 | 0.027 | - | - | 0.036 | 0.036 | - | - |
|  | **231** | 0.014 | 0.014 | 0.315 | 0.308 | 0.607 | 0.604 | 0.163 | 0.163 |
|  | **232** | - | - | 0.009 | 0.009 | - | - | - | - |
|  | **233** | 0.527 | 0.451 | 0.000 | - | - | - | 0.047 | 0.047 |
|  | **234** | 0.095 | 0.084 | 0.204 | 0.200 | 0.083 | 0.083 | - | - |
|  | **235** | 0.027 | 0.027 | - | - | - | - | - | - |
|  | **236** | 0.027 | 0.014 | 0.037 | 0.037 | 0.036 | 0.036 | - | - |
|  | **238** | 0.027 | 0.027 | 0.037 | 0.037 | 0.024 | 0.024 | - | - |
|  | **240** | - | - | 0.019 | 0.019 | 0.012 | 0.012 | - | - |
|  | **241** | 0.014 | 0.014 | - | - | - | - | - | - |
|  | **242** | 0.027 | 0.027 | - | - | - | - | 0.023 | 0.023 |
|  | **244** | - | - | - | - | - | - | 0.279 | 0.269 |
|  | **246** | 0.014 | 0.014 | - | - | - | - | - | - |
|  | **250** | 0.014 | 0.014 | - | - | - | - | 0.012 | 0.012 |
|  | **252** | 0.054 | 0.042 | - | - | - | - | 0.000 | - |
|  | **254** | 0.027 | 0.027 | - | - | - | - | 0.105 | 0.096 |
|  | **256** | 0.041 | 0.028 | - | - | - | - | - | - |
|  | **257** | - | - | 0.009 | 0.009 | - | - | - | - |
|  | **264** | - | - | - | - | 0.012 | 0.012 | - | - |
|  | **266** | - | - | - | - | 0.012 | 0.012 | - | - |
|  | **NULL** | NA | 0.134 | NA | 0.022 | NA | 0.005 | NA | 0.029 |
| **caja13** | **342** | 0.024 | 0.024 | - | - | - | - | - | - |
|  | **352** | 0.012 | 0.012 | - | - | - | - | - | - |
|  | **354** | 0.012 | 0.012 | - | - | 0.083 | 0.079 | - | - |
|  | **356** | 0.512 | 0.477 | - | - | 0.048 | 0.048 | 0.023 | 0.023 |
|  | **358** | 0.024 | 0.014 | 0.043 | 0.043 | - | - | - | - |
|  | **360** | 0.049 | 0.049 | 0.069 | 0.069 | - | - | - | - |
|  | **362** | 0.024 | 0.024 | 0.103 | 0.103 | 0.107 | 0.107 | 0.023 | 0.023 |
|  | **364** | 0.159 | 0.159 | 0.319 | 0.318 | 0.167 | 0.164 | 0.375 | 0.375 |
|  | **366** | 0.098 | 0.091 | 0.362 | 0.361 | 0.464 | 0.456 | 0.148 | 0.148 |
|  | **368** | 0.085 | 0.085 | 0.069 | 0.068 | 0.119 | 0.113 | 0.420 | 0.420 |
|  | **370** | - | - | 0.034 | 0.034 | 0.012 | 0.012 | 0.011 | 0.011 |
|  | **NULL** | NA | 0.053 | NA | 0.002 | NA | 0.021 | NA | - |
| **caja14** | **198** | - | - | 0.175 | 0.167 | 0.345 | 0.320 | 0.047 | 0.047 |
|  | **200** | - | - | 0.025 | 0.025 | 0.024 | 0.024 | - | - |
|  | **204** | 0.062 | 0.063 | 0.008 | 0.008 | 0.048 | 0.048 | - | - |
|  | **206** | 0.138 | 0.136 | - | - | 0.095 | 0.095 | 0.047 | 0.047 |
|  | **208** | 0.125 | 0.125 | 0.550 | 0.506 | 0.107 | 0.093 | 0.267 | 0.267 |
|  | **210** | 0.338 | 0.333 | 0.150 | 0.132 | 0.274 | 0.253 | 0.116 | 0.116 |
|  | **212** | 0.025 | 0.025 | 0.025 | 0.025 | 0.012 | 0.012 | 0.105 | 0.105 |
|  | **214** | 0.075 | 0.072 | 0.067 | 0.061 | 0.071 | 0.063 | 0.395 | 0.395 |
|  | **216** | - | - | - | - | 0.012 | 0.012 | 0.012 | 0.012 |
|  | **218** | 0.087 | 0.085 | - | - | - | - | 0.012 | 0.012 |
|  | **220** | - | - | - | - | 0.012 | 0.012 | - | - |
|  | **222** | 0.025 | 0.025 | - | - | - | - | - | - |
|  | **226** | 0.025 | 0.025 | - | - | - | - | - | - |
|  | **228** | 0.013 | 0.013 | - | - | - | - | - | - |
|  | **230** | 0.087 | 0.088 | - | - | - | - | - | - |
|  | **NULL** | NA | 0.012 | NA | 0.076 | NA | 0.068 | NA | - |
| **caja15** | **118** | 0.024 | 0.024 | - | - | - | - | - | - |
|  | **127** | 0.012 | 0.012 | - | - | - | - | - | - |
|  | **133** | 0.024 | 0.024 | - | - | - | - | - | - |
|  | **135** | 0.085 | 0.080 | 0.181 | 0.169 | 0.105 | 0.096 | - | - |
|  | **137** | 0.317 | 0.307 | 0.233 | 0.231 | 0.523 | 0.449 | 0.263 | 0.250 |
|  | **139** | 0.061 | 0.061 | 0.172 | 0.165 | 0.081 | 0.052 | 0.438 | 0.374 |
|  | **141** | - | - | - | - | - | - | 0.025 | 0.013 |
|  | **142** | 0.268 | 0.261 | 0.086 | 0.082 | 0.070 | 0.070 | 0.013 | 0.013 |
|  | **144** | 0.073 | 0.073 | 0.009 | 0.009 | 0.012 | 0.012 | - | - |
|  | **146** | 0.122 | 0.113 | 0.103 | 0.103 | 0.163 | 0.140 | 0.188 | 0.165 |
|  | **148** | 0.012 | 0.012 | 0.172 | 0.165 | 0.023 | 0.023 | 0.075 | 0.065 |
|  | **150** | - | - | 0.043 | 0.043 | 0.023 | 0.023 | - | - |
|  | **NULL** | NA | 0.032 | NA | 0.032 | NA | 0.135 | NA | 0.119 |
| **caja16** | **380** | - | - | 0.058 | 0.058 | - | - | - | - |
|  | **381** | - | - | 0.038 | 0.038 | - | - | 0.081 | 0.071 |
|  | **382** | - | - | - | - | 0.014 | 0.014 | 0.284 | 0.249 |
|  | **383** | - | - | 0.144 | 0.128 | 0.176 | 0.176 | - | - |
|  | **384** | 0.028 | 0.028 | 0.635 | 0.582 | 0.608 | 0.601 | 0.176 | 0.161 |
|  | **389** | 0.028 | 0.014 | 0.029 | 0.029 | 0.041 | 0.035 | 0.000 | - |
|  | **391** | 0.014 | 0.014 | 0.096 | 0.083 | 0.108 | 0.108 | 0.446 | 0.413 |
|  | **393** | 0.097 | 0.075 | - | - | 0.027 | 0.027 | - | - |
|  | **395** | 0.222 | 0.177 | - | - | - | - | - | - |
|  | **397** | 0.167 | 0.137 | - | - | 0.027 | 0.027 | - | - |
|  | **399** | 0.347 | 0.304 | - | - | - | - | - | - |
|  | **401** | 0.014 | 0.014 | - | - | - | - | - | - |
|  | **403** | 0.083 | 0.072 | - | - | - | - | - | - |
|  | **406** | - | - | - | - | - | - | 0.014 | 0.014 |
|  | **NULL** | NA | 0.166 | NA | 0.081 | NA | 0.013 | NA | 0.092 |
| **caja17** | **352** | 0.038 | 0.038 | - | - | - | - | - | - |
|  | **358** | 0.128 | 0.122 | - | - | - | - | - | - |
|  | **360** | 0.013 | 0.013 | - | - | - | - | 0.091 | 0.089 |
|  | **362** | 0.128 | 0.110 | 0.017 | 0.017 | 0.024 | 0.024 | 0.159 | 0.158 |
|  | **364** | - | - | 0.092 | 0.091 | 0.083 | 0.076 | - | - |
|  | **366** | 0.141 | 0.136 | 0.600 | 0.599 | 0.012 | 0.012 | 0.057 | 0.057 |
|  | **368** | 0.051 | 0.042 | 0.033 | 0.033 | 0.190 | 0.176 | 0.102 | 0.100 |
|  | **370** | 0.051 | 0.051 | 0.033 | 0.033 | 0.107 | 0.101 | - | - |
|  | **372** | 0.051 | 0.051 | 0.083 | 0.083 | 0.262 | 0.246 | - | - |
|  | **374** | 0.141 | 0.130 | 0.017 | 0.017 | 0.024 | 0.024 | - | - |
|  | **376** | 0.077 | 0.077 | 0.083 | 0.083 | 0.119 | 0.113 | 0.045 | 0.045 |
|  | **378** | 0.038 | 0.038 | 0.025 | 0.025 | 0.167 | 0.156 | 0.364 | 0.359 |
|  | **380** | 0.038 | 0.038 | - | - | - | - | - | - |
|  | **382** | - | - | - | - | 0.012 | 0.012 | - | - |
|  | **386** | - | - | - | - | - | - | 0.102 | 0.102 |
|  | **388** | 0.026 | 0.026 | - | - | - | - | - | - |
|  | **396** | 0.013 | 0.013 | - | - | - | - | - | - |
|  | **398** | 0.038 | 0.038 | 0.008 | 0.008 | - | - | - | - |
|  | **400** | - | - | 0.008 | 0.008 | - | - | - | - |
|  | **403** | 0.013 | 0.013 | - | - | - | - | - | - |
|  | **418** | - | - | - | - | - | - | 0.080 | 0.080 |
|  | **426** | 0.013 | 0.013 | - | - | - | - | - | - |
|  | **NULL** | NA | 0.049 | NA | 0.002 | NA | 0.061 | NA | 0.011 |
| **caja18** | **297** | 0.176 | 0.175 | 0.009 | 0.009 | - | - | - | - |
|  | **299** | 0.108 | 0.107 | 0.038 | 0.038 | 0.012 | 0.012 | 0.267 | 0.246 |
|  | **301** | 0.365 | 0.363 | 0.425 | 0.382 | 0.634 | 0.586 | 0.078 | 0.070 |
|  | **303** | 0.068 | 0.065 | 0.255 | 0.211 | 0.061 | 0.061 | 0.489 | 0.457 |
|  | **305** | 0.027 | 0.027 | 0.274 | 0.246 | 0.049 | 0.049 | 0.133 | 0.121 |
|  | **307** | 0.041 | 0.041 | - | - | 0.134 | 0.114 | 0.022 | 0.022 |
|  | **309** | 0.081 | 0.081 | - | - | 0.061 | 0.061 | - | - |
|  | **311** | 0.135 | 0.135 | - | - | 0.012 | 0.012 | 0.011 | 0.011 |
|  | **315** | - | - | - | - | 0.037 | 0.037 | - | - |
|  | **NULL** | NA | 0.007 | NA | 0.113 | NA | 0.069 | NA | 0.072 |
| **caja19** | **320** | 0.037 | 0.038 | - | - | - | - | 0.062 | 0.053 |
|  | **322** | 0.062 | 0.041 | 0.067 | 0.067 | 0.189 | 0.137 | 0.062 | 0.063 |
|  | **324** | - | - | 0.202 | 0.181 | - | - | - | - |
|  | **344** | - | - | 0.010 | 0.010 | - | - | - | - |
|  | **346** | 0.350 | 0.294 | - | - | - | - | - | - |
|  | **348** | 0.287 | 0.239 | - | - | - | - | - | - |
|  | **350** | 0.125 | 0.116 | 0.010 | - | 0.014 | 0.014 | - | - |
|  | **352** | 0.037 | 0.038 | - | - | 0.135 | 0.090 | 0.812 | 0.749 |
|  | **354** | 0.050 | 0.039 | 0.442 | 0.414 | 0.662 | 0.531 | 0.050 | 0.040 |
|  | **356** | - | - | 0.019 | 0.019 | - | - | - | - |
|  | **358** | - | - | 0.067 | 0.067 | - | - | - | - |
|  | **365** | - | - | 0.163 | 0.145 | - | - | - | - |
|  | **368** | - | - | 0.010 | 0.010 | - | - | 0.013 | 0.013 |
|  | **372** | 0.013 | 0.013 | - | - | - | - | - | - |
|  | **373** | - | - | 0.010 | 0.010 | - | - | - | - |
|  | **376** | 0.013 | 0.013 | - | - | - | - | - | - |
|  | **378** | 0.025 | 0.025 | - | - | - | - | - | - |
|  | **NULL** | NA | 0.146 | NA | 0.067 | NA | 0.228 | NA | 0.083 |
| **cj1** | **117** | 0.090 | 0.082 | 0.640 | 0.640 | 0.524 | 0.446 | 0.430 | 0.422 |
|  | **119** | - | - | - | - | - | - | 0.023 | 0.023 |
|  | **125** | 0.090 | 0.082 | - | - | - | - | - | - |
|  | **129** | 0.103 | 0.088 | - | - | 0.037 | 0.025 | 0.000 | - |
|  | **132** | 0.128 | 0.122 | - | - | 0.012 | 0.012 | 0.326 | 0.318 |
|  | **133** | - | - | - | - | 0.012 | 0.012 | - | - |
|  | **134** | 0.064 | 0.064 | - | - | - | - | - | - |
|  | **135** | - | - | 0.035 | 0.035 | - | - | - | - |
|  | **136** | 0.013 | 0.013 | - | - | - | - | - | - |
|  | **137** | 0.038 | 0.038 | 0.044 | 0.044 | - | - | - | - |
|  | **139** | 0.064 | 0.055 | 0.211 | 0.211 | 0.268 | 0.219 | 0.035 | 0.035 |
|  | **141** | 0.051 | 0.051 | 0.026 | 0.026 | - | - | - | - |
|  | **143** | 0.013 | 0.013 | - | - | - | - | - | - |
|  | **144** | 0.064 | 0.064 | 0.026 | 0.026 | 0.073 | 0.073 | - | - |
|  | **146** | 0.192 | 0.177 | 0.009 | 0.009 | 0.061 | 0.051 | - | - |
|  | **148** | 0.038 | 0.038 | - | - | - | - | - | - |
|  | **149** | 0.026 | 0.026 | - | - | - | - | - | - |
|  | **150** | 0.026 | 0.026 | - | - | - | - | - | - |
|  | **152** | - | - | - | - | 0.012 | 0.012 | 0.186 | 0.182 |
|  | **177** | - | - | 0.009 | 0.009 | - | - | - | - |
|  | **NULL** | NA | 0.061 | NA | - | NA | 0.149 | NA | 0.020 |
| **cj6** | **127** | 0.061 | 0.043 | - | - | - | - | 0.035 | 0.035 |
|  | **135** | 0.073 | 0.057 | 0.093 | 0.088 | 0.637 | 0.605 | 0.035 | 0.035 |
|  | **137** | 0.024 | 0.024 | 0.042 | 0.036 | 0.037 | 0.038 | 0.384 | 0.355 |
|  | **139** | 0.085 | 0.085 | 0.025 | 0.025 | 0.037 | 0.038 | 0.430 | 0.391 |
|  | **141** | 0.232 | 0.223 | 0.102 | 0.092 | 0.025 | 0.025 | 0.035 | 0.035 |
|  | **143** | 0.159 | 0.148 | 0.661 | 0.615 | 0.263 | 0.244 | 0.023 | 0.023 |
|  | **145** | 0.183 | 0.178 | 0.051 | 0.051 | - | - | 0.012 | 0.012 |
|  | **147** | 0.037 | 0.037 | 0.017 | 0.017 | - | - | - | - |
|  | **149** | 0.073 | 0.073 | 0.008 | 0.008 | - | - | - | - |
|  | **151** | 0.012 | 0.012 | - | - | - | - | - | - |
|  | **153** | 0.037 | 0.037 | - | - | - | - | 0.047 | 0.047 |
|  | **157** | 0.024 | 0.024 | - | - | - | - | - | - |
|  | **NULL** | NA | 0.058 | NA | 0.068 | NA | 0.050 | NA | 0.068 |
| **cj11** | **114** | - | - | - | - | 0.013 | 0.013 | - | - |
|  | **116** | 0.268 | 0.212 | 0.365 | 0.357 | 0.250 | 0.215 | 0.756 | 0.756 |
|  | **118** | 0.183 | 0.137 | 0.594 | 0.579 | 0.688 | 0.598 | 0.061 | 0.061 |
|  | **120** | 0.524 | 0.423 | 0.042 | 0.035 | 0.050 | 0.039 | 0.183 | 0.183 |
|  | **124** | 0.012 | 0.012 | - | - | - | - | - | - |
|  | **130** | 0.012 | 0.012 | - | - | - | - | - | - |
|  | **NULL** | NA | 0.203 | NA | 0.029 | NA | 0.135 | NA | - |
| **cj14** | **121** | 0.474 | 0.426 | - | - | 0.037 | 0.038 | - | - |
|  | **150** | 0.026 | 0.026 | - | - | 0.013 | 0.013 | 0.100 | 0.098 |
|  | **152** | - | - | 0.010 | 0.010 | - | - | 0.156 | 0.156 |
|  | **154** | - | - | 0.176 | 0.173 | - | - | - | - |
|  | **156** | 0.013 | 0.013 | 0.353 | 0.340 | 0.312 | 0.303 | 0.211 | 0.210 |
|  | **158** | 0.026 | 0.026 | 0.039 | 0.032 | 0.025 | 0.025 | - | - |
|  | **160** | 0.077 | 0.077 | 0.157 | 0.154 | 0.400 | 0.393 | 0.167 | 0.165 |
|  | **162** | - | - | 0.020 | 0.020 | 0.062 | 0.063 | 0.011 | 0.011 |
|  | **164** | 0.038 | 0.038 | 0.039 | 0.039 | 0.037 | 0.038 | 0.033 | 0.033 |
|  | **166** | 0.064 | 0.054 | - | - | 0.013 | 0.013 | 0.011 | 0.011 |
|  | **168** | 0.038 | 0.027 | - | - | 0.013 | 0.013 | 0.056 | 0.052 |
|  | **169** | - | - | - | - | 0.037 | 0.038 | - | - |
|  | **170** | 0.090 | 0.072 | 0.176 | 0.164 | - | - | 0.244 | 0.240 |
|  | **172** | 0.013 | 0.013 | - | - | 0.013 | 0.013 | - | - |
|  | **174** | 0.077 | 0.077 | 0.020 | 0.020 | 0.025 | 0.015 | 0.011 | 0.011 |
|  | **176** | 0.051 | 0.051 | 0.010 | 0.010 | 0.013 | 0.013 | - | - |
|  | **180** | 0.013 | 0.013 | - | - | - | - | - | - |
|  | **NULL** | NA | 0.088 | NA | 0.038 | NA | 0.026 | NA | 0.014 |
| **ham1** | **175** | - | - | - | - | - | - | 0.293 | 0.283 |
|  | **177** | 0.038 | 0.028 | - | - | - | - | 0.049 | 0.049 |
|  | **181** | 0.038 | 0.038 | - | - | 0.013 | 0.013 | - | - |
|  | **183** | 0.013 | 0.013 | 0.114 | 0.114 | 0.438 | 0.421 | - | - |
|  | **185** | - | - | - | - | 0.037 | 0.029 | 0.110 | 0.110 |
|  | **187** | 0.128 | 0.121 | 0.018 | 0.018 | 0.025 | 0.025 | 0.024 | 0.024 |
|  | **188** | - | - | - | - | 0.013 | 0.013 | - | - |
|  | **189** | 0.192 | 0.181 | 0.123 | 0.123 | 0.025 | 0.025 | - | - |
|  | **191** | 0.269 | 0.251 | 0.193 | 0.193 | 0.200 | 0.190 | 0.110 | 0.110 |
|  | **193** | 0.167 | 0.161 | 0.219 | 0.219 | 0.087 | 0.088 | 0.024 | 0.024 |
|  | **195** | 0.103 | 0.087 | 0.237 | 0.237 | 0.062 | 0.063 | 0.390 | 0.382 |
|  | **197** | 0.038 | 0.038 | 0.079 | 0.079 | - | - | - | - |
|  | **199** | 0.013 | 0.013 | 0.009 | 0.009 | 0.100 | 0.100 | - | - |
|  | **201** | - | - | 0.009 | 0.009 | - | - | - | - |
|  | **NULL** | NA | 0.068 | - | - | NA | 0.035 | NA | - |
| **ham3** | **74** | - | - | - | - | 0.013 | 0.013 | - | - |
|  | **78** | - | - | - | - | 0.013 | 0.013 | - | - |
|  | **84** | - | - | 0.017 | 0.017 | - | - | - | - |
|  | **86** | 0.038 | 0.038 | 0.017 | 0.017 | - | - | 0.012 | 0.012 |
|  | **88** | - | - | - | - | 0.079 | 0.079 | 0.107 | 0.105 |
|  | **90** | 0.077 | 0.072 | 0.025 | 0.019 | 0.039 | 0.039 | 0.405 | 0.399 |
|  | **92** | 0.115 | 0.115 | 0.042 | 0.042 | 0.053 | 0.053 | 0.036 | 0.036 |
|  | **94** | 0.103 | 0.103 | 0.288 | 0.279 | 0.316 | 0.302 | 0.214 | 0.213 |
|  | **96** | 0.295 | 0.290 | 0.483 | 0.465 | 0.289 | 0.279 | 0.095 | 0.093 |
|  | **98** | 0.077 | 0.077 | 0.051 | 0.051 | 0.171 | 0.144 | - | - |
|  | **100** | 0.077 | 0.072 | 0.068 | 0.063 | - | - | - | - |
|  | **102** | 0.051 | 0.051 | 0.008 | 0.008 | 0.026 | 0.026 | 0.048 | 0.048 |
|  | **104** | 0.051 | 0.045 | - | - | - | - | - | - |
|  | **106** | 0.026 | 0.026 | - | - | - | - | 0.083 | 0.083 |
|  | **108** | 0.051 | 0.051 | - | - | - | - | - | - |
|  | **111** | 0.038 | 0.038 | - | - | - | - | - | - |
|  | **NULL** | NA | 0.019 | NA | 0.039 | NA | 0.052 | NA | 0.012 |
| **ham8** | **270** | 0.016 | 0.016 | - | - | - | - | - | - |
|  | **272** | 0.016 | 0.016 | 0.009 | 0.009 | 0.013 | 0.013 | 0.167 | 0.164 |
|  | **273** | - | - | - | - | - | - | 0.069 | 0.064 |
|  | **274** | 0.194 | 0.161 | - | - | 0.013 | 0.013 | 0.083 | 0.083 |
|  | **276** | - | - | - | - | - | - | 0.208 | 0.201 |
|  | **280** | 0.032 | 0.032 | - | - | - | - | - | - |
|  | **281** | 0.065 | 0.065 | - | - | 0.026 | 0.026 | - | - |
|  | **282** | 0.016 | 0.016 | 0.113 | 0.112 | 0.090 | 0.090 | - | - |
|  | **283** | 0.048 | 0.048 | - | - | 0.077 | 0.061 | - | - |
|  | **284** | - | - | 0.085 | 0.085 | 0.077 | 0.077 | 0.153 | 0.153 |
|  | **285** | 0.065 | 0.065 | - | - | - | - | - | - |
|  | **286** | 0.065 | 0.052 | 0.660 | 0.657 | 0.526 | 0.498 | 0.264 | 0.258 |
|  | **287** | 0.258 | 0.231 | 0.009 | 0.009 | 0.013 | 0.013 | 0.014 | 0.014 |
|  | **288** | - | - | 0.104 | 0.103 | - | - | - | - |
|  | **289** | 0.097 | 0.086 | 0.009 | 0.009 | 0.026 | 0.026 | 0.014 | 0.014 |
|  | **290** | - | - | 0.009 | 0.009 | 0.128 | 0.123 | 0.028 | 0.028 |
|  | **291** | 0.065 | 0.065 | - | - | - | - | - | - |
|  | **292** | - | - | - | - | 0.013 | 0.013 | - | - |
|  | **293** | 0.065 | 0.065 | - | - | - | - | - | - |
|  | **NULL** | NA | 0.082 | NA | 0.005 | NA | 0.049 | NA | 0.023 |
| **ham26** | **163** | - | - | 0.018 | 0.011 | - | - | - | - |
|  | **165** | 0.033 | 0.033 | 0.430 | 0.426 | 0.012 | 0.012 | 0.267 | 0.243 |
|  | **167** | - | - | 0.018 | 0.018 | - | - | 0.012 | 0.012 |
|  | **168** | - | - | - | - | 0.071 | 0.070 | - | - |
|  | **170** | 0.517 | 0.425 | - | - | - | - | - | - |
|  | **172** | 0.100 | 0.100 | - | - | - | - | 0.023 | 0.023 |
|  | **174** | - | - | - | - | 0.036 | 0.036 | 0.012 | 0.012 |
|  | **176** | 0.017 | 0.017 | 0.026 | 0.026 | 0.024 | 0.024 | 0.198 | 0.189 |
|  | **178** | - | - | 0.070 | 0.070 | 0.024 | 0.024 | - | - |
|  | **180** | 0.183 | 0.161 | 0.193 | 0.192 | 0.298 | 0.297 | 0.256 | 0.249 |
|  | **182** | 0.100 | 0.073 | 0.211 | 0.209 | 0.202 | 0.201 | 0.047 | 0.038 |
|  | **184** | 0.050 | 0.035 | 0.026 | 0.026 | 0.321 | 0.320 | 0.186 | 0.182 |
|  | **186** | - | - | 0.009 | 0.009 | 0.012 | 0.012 | - | - |
|  | **NULL** | NA | 0.155 | NA | 0.013 | NA | 0.005 | NA | 0.053 |
| **ham30** | **284** | 0.012 | 0.012 | - | - | - | - | - | - |
|  | **286** | 0.012 | 0.012 | 0.036 | 0.030 | 0.037 | 0.035 | 0.302 | 0.278 |
|  | **288** | 0.183 | 0.165 | 0.018 | 0.018 | 0.175 | 0.175 | 0.047 | 0.038 |
|  | **290** | 0.341 | 0.317 | 0.134 | 0.128 | 0.275 | 0.275 | 0.198 | 0.190 |
|  | **292** | 0.354 | 0.317 | 0.045 | 0.045 | 0.025 | 0.025 | 0.302 | 0.293 |
|  | **294** | 0.024 | 0.024 | 0.188 | 0.185 | 0.287 | 0.287 | 0.012 | 0.012 |
|  | **296** | 0.037 | 0.037 | 0.062 | 0.063 | - | - | 0.140 | 0.140 |
|  | **298** | 0.024 | 0.024 | 0.455 | 0.439 | 0.150 | 0.149 | - | - |
|  | **300** | 0.012 | 0.012 | 0.054 | 0.054 | 0.013 | 0.013 | - | - |
|  | **302** | - | - | - | - | 0.037 | 0.038 | - | - |
|  | **309** | - | - | 0.009 | 0.009 | - | - | - | - |
|  | **NULL** | NA | 0.078 | NA | 0.030 | NA | 0.004 | NA | 0.050 |
| **ham38** | **257** | 0.026 | 0.026 | - | - | - | - | - | - |
|  | **260** | 0.013 | 0.013 | - | - | - | - | - | - |
|  | **264** | 0.092 | 0.083 | - | - | - | - | 0.143 | 0.129 |
|  | **266** | 0.026 | 0.026 | - | - | - | - | - | - |
|  | **268** | 0.382 | 0.360 | - | - | 0.026 | 0.014 | - | - |
|  | **269** | 0.013 | 0.013 | - | - | - | - | - | - |
|  | **270** | 0.105 | 0.105 | 0.067 | 0.055 | 0.244 | 0.224 | 0.155 | 0.148 |
|  | **272** | 0.145 | 0.130 | 0.225 | 0.216 | 0.013 | 0.013 | 0.036 | 0.036 |
|  | **274** | 0.013 | 0.013 | 0.525 | 0.497 | 0.372 | 0.317 | - | - |
|  | **275** | - | - | 0.017 | 0.017 | 0.038 | 0.027 | 0.202 | 0.179 |
|  | **276** | - | - | 0.008 | 0.008 | - | - | 0.012 | 0.012 |
|  | **277** | 0.026 | 0.014 | 0.008 | 0.008 | 0.077 | 0.077 | 0.119 | 0.112 |
|  | **278** | - | - | 0.017 | 0.009 | 0.013 | 0.013 | 0.012 | 0.012 |
|  | **279** | 0.066 | 0.056 | 0.042 | 0.042 | 0.103 | 0.093 | 0.048 | 0.048 |
|  | **281** | 0.079 | 0.059 | 0.092 | 0.087 | 0.051 | 0.040 | 0.107 | 0.092 |
|  | **283** | 0.013 | 0.013 | - | - | 0.013 | 0.013 | - | - |
|  | **285** | - | - | - | - | 0.051 | 0.051 | 0.167 | 0.148 |
|  | **NULL** | NA | 0.087 | NA | 0.061 | NA | 0.118 | NA | 0.085 |
| **ham47** | **278** | - | - | 0.009 | 0.009 | - | - | - | - |
|  | **281** | - | - | 0.231 | 0.231 | - | - | - | - |
|  | **283** | 0.050 | 0.050 | 0.028 | 0.028 | 0.037 | 0.037 | 0.151 | 0.140 |
|  | **285** | 0.062 | 0.053 | 0.065 | 0.064 | 0.220 | 0.216 | 0.209 | 0.203 |
|  | **287** | 0.062 | 0.053 | 0.037 | 0.037 | 0.159 | 0.159 | 0.337 | 0.323 |
|  | **289** | 0.150 | 0.144 | 0.574 | 0.573 | 0.354 | 0.350 | 0.221 | 0.218 |
|  | **291** | 0.400 | 0.380 | 0.019 | 0.019 | 0.122 | 0.122 | 0.035 | 0.035 |
|  | **293** | 0.212 | 0.197 | 0.019 | 0.019 | 0.061 | 0.061 | - | - |
|  | **295** | 0.037 | 0.027 | 0.019 | 0.019 | 0.049 | 0.045 | 0.023 | 0.023 |
|  | **300** | 0.013 | 0.013 | - | - | - | - | 0.023 | 0.023 |
|  | **302** | 0.013 | 0.013 | - | - | - | - | - | - |
|  | **NULL** | NA | 0.071 | NA | 0.003 | NA | 0.011 | NA | 0.035 |
| **ham55** | **243** | 0.550 | 0.472 | - | - | - | - | - | - |
|  | **245** | 0.050 | 0.039 | - | - | - | - | - | - |
|  | **255** | 0.163 | 0.155 | 0.018 | 0.018 | - | - | - | - |
|  | **259** | - | - | - | - | 0.014 | 0.014 | 0.149 | 0.149 |
|  | **261** | 0.013 | 0.013 | - | - | - | - | - | - |
|  | **264** | - | - | 0.136 | 0.136 | - | - | - | - |
|  | **266** | - | - | 0.009 | 0.009 | - | - | 0.027 | 0.027 |
|  | **268** | 0.025 | 0.025 | 0.409 | 0.409 | 0.542 | 0.511 | 0.068 | 0.068 |
|  | **270** | 0.062 | 0.063 | 0.018 | 0.018 | 0.194 | 0.184 | 0.041 | 0.041 |
|  | **272** | 0.037 | 0.038 | 0.009 | 0.009 | 0.111 | 0.111 | - | - |
|  | **274** | 0.037 | 0.026 | 0.373 | 0.373 | 0.028 | 0.028 | 0.514 | 0.514 |
|  | **276** | 0.013 | 0.013 | 0.018 | 0.018 | 0.097 | 0.081 | - | - |
|  | **277** | - | - | - | - | 0.014 | 0.014 | - | - |
|  | **278** | 0.013 | 0.013 | - | - | - | - | - | - |
|  | **287** | - | - | 0.009 | 0.009 | - | - | - | - |
|  | **289** | 0.025 | 0.025 | - | - | - | - | 0.095 | 0.095 |
|  | **297** | 0.013 | 0.013 | - | - | - | - | - | - |
|  | **302** | - | - | - | - | - | - | 0.014 | 0.014 |
|  | **304** | - | - | - | - | - | - | 0.095 | 0.095 |
|  | **NULL** | NA | 0.107 | NA | - | NA | 0.058 | NA | - |
| **ham57** | **230** | 0.061 | 0.061 | - | - | - | - | 0.352 | 0.350 |
|  | **232** | 0.073 | 0.064 | 0.017 | 0.017 | 0.110 | 0.095 | 0.091 | 0.091 |
|  | **234** | 0.524 | 0.481 | 0.322 | 0.309 | 0.585 | 0.547 | 0.227 | 0.226 |
|  | **236** | 0.232 | 0.221 | 0.610 | 0.587 | 0.244 | 0.225 | 0.136 | 0.134 |
|  | **238** | - | - | 0.034 | 0.034 | 0.012 | 0.012 | 0.159 | 0.158 |
|  | **240** | - | - | 0.017 | 0.017 | 0.049 | 0.049 | 0.034 | 0.034 |
|  | **242** | 0.085 | 0.058 | - | - | - | - | - | - |
|  | **244** | 0.024 | 0.024 | - | - | - | - | - | - |
|  | **NULL** | NA | 0.092 | NA | 0.036 | NA | 0.071 | NA | 0.007 |
| **ham60** | **120** | - | - | - | - | - | - | 0.310 | 0.293 |
|  | **122** | 0.013 | 0.013 | - | - | - | - | - | - |
|  | **128** | 0.256 | 0.218 | - | - | - | - | - | - |
|  | **130** | 0.423 | 0.362 | 0.008 | 0.008 | 0.013 | 0.013 | 0.190 | 0.178 |
|  | **132** | 0.128 | 0.099 | 0.763 | 0.689 | 0.487 | 0.435 | 0.321 | 0.305 |
|  | **134** | 0.013 | 0.013 | 0.034 | 0.026 | 0.013 | 0.013 | - | - |
|  | **136** | - | - | 0.017 | 0.017 | 0.138 | 0.121 | 0.071 | 0.071 |
|  | **138** | 0.077 | 0.043 | 0.017 | 0.009 | - | - | 0.036 | 0.036 |
|  | **140** | 0.026 | 0.026 | 0.017 | 0.017 | 0.062 | 0.052 | - | - |
|  | **142** | 0.064 | 0.064 | 0.136 | 0.119 | 0.225 | 0.198 | 0.071 | 0.071 |
|  | **146** | - | - | 0.008 | 0.008 | 0.062 | 0.052 | - | - |
|  | **NULL** | NA | 0.162 | NA | 0.105 | NA | 0.116 | NA | 0.045 |
| **ham79** | **123** | - | - | - | - | - | - | 0.064 | 0.054 |
|  | **125** | 0.071 | 0.051 | 0.176 | 0.172 | 0.300 | 0.283 | - | - |
|  | **127** | 0.029 | 0.016 | 0.118 | 0.112 | 0.500 | 0.478 | 0.192 | 0.153 |
|  | **129** | 0.057 | 0.057 | 0.373 | 0.349 | 0.186 | 0.181 | 0.103 | 0.103 |
|  | **131** | 0.086 | 0.086 | 0.088 | 0.069 | - | - | 0.462 | 0.399 |
|  | **133** | 0.443 | 0.418 | 0.167 | 0.162 | 0.014 | 0.014 | 0.013 | 0.013 |
|  | **135** | 0.143 | 0.143 | 0.039 | 0.039 | - | - | - | - |
|  | **137** | 0.071 | 0.071 | 0.039 | 0.031 | - | - | 0.141 | 0.133 |
|  | **139** | 0.029 | 0.029 | - | - | - | - | - | - |
|  | **141** | 0.071 | 0.062 | - | - | - | - | 0.026 | 0.026 |
|  | **NULL** | N/A | 0.068 | NA | 0.065 | NA | 0.043 | NA | 0.121 |
| **ham91** | **128** | 0.012 | 0.012 | - | - | 0.012 | 0.012 | - | - |
|  | **134** | 0.012 | 0.012 | - | - | - | - | 0.047 | 0.047 |
|  | **136** | 0.073 | 0.067 | 0.025 | 0.025 | 0.070 | 0.070 | - | - |
|  | **138** | 0.305 | 0.297 | 0.008 | 0.008 | 0.012 | 0.012 | 0.302 | 0.295 |
|  | **140** | 0.195 | 0.188 | 0.213 | 0.210 | 0.047 | 0.040 | 0.186 | 0.180 |
|  | **142** | 0.122 | 0.112 | 0.492 | 0.477 | 0.640 | 0.623 | 0.198 | 0.196 |
|  | **144** | 0.159 | 0.159 | 0.033 | 0.027 | 0.023 | 0.023 | - | - |
|  | **146** | 0.012 | 0.012 | 0.139 | 0.139 | 0.116 | 0.116 | - | - |
|  | **148** | 0.024 | 0.024 | 0.008 | 0.008 | 0.012 | 0.012 | - | - |
|  | **150** | 0.012 | 0.012 | 0.049 | 0.045 | 0.023 | 0.023 | - | - |
|  | **152** | 0.012 | 0.012 | 0.008 | 0.008 | - | - | 0.116 | 0.113 |
|  | **154** | 0.012 | 0.012 | 0.008 | 0.008 | 0.035 | 0.035 | 0.151 | 0.151 |
|  | **156** | 0.049 | 0.041 | - | - | - | - | - | - |
|  | **160** | - | - | 0.016 | 0.016 | 0.012 | 0.012 | - | - |
|  | **NULL** | NA | 0.040 | NA | 0.029 | NA | 0.023 | NA | 0.018 |
| **ham96** | **326** | 0.221 | 0.221 | - | - | - | - | - | - |
|  | **328** | 0.044 | 0.034 | - | - | - | - | - | - |
|  | **330** | - | - | 0.052 | 0.052 | 0.264 | 0.263 | 0.013 | 0.013 |
|  | **332** | - | - | 0.405 | 0.405 | 0.097 | 0.095 | - | - |
|  | **334** | 0.029 | 0.029 | 0.414 | 0.414 | 0.361 | 0.358 | 0.037 | 0.038 |
|  | **336** | 0.279 | 0.268 | 0.026 | 0.026 | 0.181 | 0.178 | 0.150 | 0.141 |
|  | **338** | 0.029 | 0.029 | 0.009 | 0.009 | - | - | 0.537 | 0.515 |
|  | **340** | - | - | 0.052 | 0.052 | 0.014 | 0.014 | 0.050 | 0.050 |
|  | **342** | 0.044 | 0.044 | 0.034 | 0.034 | 0.014 | 0.014 | 0.212 | 0.206 |
|  | **344** | 0.118 | 0.112 | - | - | - | - | - | - |
|  | **346** | 0.059 | 0.059 | 0.009 | 0.009 | 0.028 | 0.028 | - | - |
|  | **350** | - | - | - | - | 0.014 | 0.014 | - | - |
|  | **352** | - | - | - | - | 0.028 | 0.028 | - | - |
|  | **356** | 0.088 | 0.082 | - | - | - | - | - | - |
|  | **362** | 0.015 | 0.015 | - | - | - | - | - | - |
|  | **368** | 0.015 | 0.015 | - | - | - | - | - | - |
|  | **370** | 0.029 | 0.029 | - | - | - | - | - | - |
|  | **372** | 0.029 | 0.029 | - | - | - | - | - | - |
|  | **NULL** | NA | 0.034 | NA | - | NA | 0.009 | NA | 0.038 |
| **ham100** | **220** | 0.037 | 0.026 | - | - | - | - | 0.116 | 0.116 |
|  | **222** | 0.025 | 0.025 | - | - | - | - | - | - |
|  | **224** | 0.113 | 0.103 | - | - | - | - | - | - |
|  | **226** | 0.100 | 0.090 | - | - | 0.027 | 0.027 | 0.477 | 0.453 |
|  | **228** | - | - | 0.018 | 0.018 | - | - | - | - |
|  | **230** | 0.100 | 0.070 | 0.202 | 0.202 | 0.162 | 0.154 | 0.023 | 0.023 |
|  | **232** | 0.025 | 0.025 | - | - | 0.014 | 0.014 | - | - |
|  | **234** | 0.050 | 0.050 | - | - | 0.054 | 0.054 | - | - |
|  | **236** | 0.212 | 0.173 | 0.158 | 0.158 | 0.162 | 0.158 | 0.209 | 0.187 |
|  | **238** | 0.237 | 0.192 | 0.167 | 0.167 | 0.014 | 0.014 | 0.116 | 0.104 |
|  | **240** | 0.062 | 0.063 | 0.211 | 0.211 | 0.365 | 0.356 | 0.058 | 0.058 |
|  | **242** | - | - | 0.070 | 0.070 | - | - | - | - |
|  | **244** | 0.037 | 0.038 | 0.149 | 0.149 | 0.081 | 0.067 | - | - |
|  | **246** | - | - | 0.026 | 0.026 | - | - | - | - |
|  | **248** | - | - | - | - | 0.122 | 0.122 | - | - |
|  | **NULL** | NA | 0.144 | NA | - | NA | 0.036 | NA | 0.059 |
| **ham101** | **262** | 0.316 | 0.275 | 0.104 | 0.102 | 0.146 | 0.024 | 0.384 | 0.324 |
|  | **268** | 0.105 | 0.095 | 0.236 | 0.234 | 0.293 | 0.104 | 0.198 | 0.158 |
|  | **270** | 0.053 | 0.041 | 0.009 | 0.009 | - | - | - | - |
|  | **272** | 0.237 | 0.198 | - | - | 0.024 | 0.276 | - | - |
|  | **274** | - | - | 0.019 | 0.019 | 0.012 | 0.142 | 0.023 | 0.023 |
|  | **276** | 0.158 | 0.149 | 0.019 | 0.019 | 0.037 | 0.340 | 0.105 | 0.105 |
|  | **278** | 0.053 | 0.041 | 0.142 | 0.142 | 0.110 | 0.037 | 0.093 | 0.064 |
|  | **280** | 0.066 | 0.043 | 0.396 | 0.393 | 0.354 | 0.024 | 0.186 | 0.145 |
|  | **282** | 0.013 | 0.013 | 0.047 | 0.047 | 0.024 | 0.012 | - | - |
|  | **284** | - | - | 0.028 | 0.024 | - | - | 0.012 | 0.012 |
|  | **NULL** | NA | 0.144 | NA | 0.010 | NA | 0.040 | NA | 0.169 |
| **ham102** | **162** | 0.028 | 0.028 | - | - | 0.098 | 0.093 | 0.028 | 0.028 |
|  | **164** | 0.042 | 0.042 | - | - | - | - | 0.028 | 0.028 |
|  | **166** | 0.014 | 0.014 | - | - | 0.037 | 0.037 | 0.250 | 0.242 |
|  | **168** | 0.097 | 0.097 | 0.018 | 0.018 | 0.305 | 0.295 | 0.306 | 0.294 |
|  | **170** | 0.069 | 0.061 | 0.418 | 0.400 | 0.049 | 0.049 | - | - |
|  | **172** | 0.028 | 0.028 | 0.445 | 0.425 | 0.232 | 0.227 | - | - |
|  | **174** | 0.153 | 0.136 | 0.082 | 0.064 | 0.207 | 0.205 | 0.236 | 0.230 |
|  | **176** | 0.056 | 0.056 | 0.036 | 0.036 | 0.061 | 0.055 | - | - |
|  | **178** | 0.208 | 0.200 | - | - | 0.012 | 0.012 | - | - |
|  | **180** | 0.139 | 0.127 | - | - | - | - | 0.139 | 0.135 |
|  | **182** | 0.083 | 0.083 | - | - | - | - | 0.014 | 0.014 |
|  | **184** | 0.083 | 0.083 | - | - | - | - | - | - |
|  | **NULL** | NA | 0.045 | NA | 0.056 | NA | 0.028 | NA | 0.030 |
| **ham103** | **89** | - | - | 0.009 | 0.009 | - | - | 0.500 | 0.430 |
|  | **91** | 0.100 | 0.077 | - | - | 0.103 | 0.056 | 0.059 | 0.046 |
|  | **93** | 0.414 | 0.347 | - | - | 0.034 | 0.018 | 0.103 | 0.091 |
|  | **97** | 0.014 | 0.014 | - | - | - | - | - | - |
|  | **100** | 0.029 | 0.029 | - | - | - | - | - | - |
|  | **104** | - | - | 0.018 | 0.018 | - | - | - | - |
|  | **106** | 0.014 | 0.014 | 0.009 | 0.009 | - | - | - | - |
|  | **108** | - | - | 0.018 | 0.018 | - | - | - | - |
|  | **110** | - | - | 0.036 | 0.036 | 0.017 | 0.017 | - | - |
|  | **112** | 0.171 | 0.152 | 0.082 | 0.082 | - | - | - | - |
|  | **114** | 0.014 | 0.014 | 0.091 | 0.087 | 0.276 | 0.213 | 0.176 | 0.157 |
|  | **116** | 0.043 | 0.043 | 0.400 | 0.395 | 0.086 | 0.055 | 0.088 | 0.064 |
|  | **118** | 0.129 | 0.096 | 0.118 | 0.117 | 0.345 | 0.262 | - | - |
|  | **120** | 0.057 | 0.045 | 0.200 | 0.198 | 0.103 | 0.056 | 0.074 | 0.048 |
|  | **122** | - | - | 0.009 | 0.009 | 0.017 | 0.017 | - | - |
|  | **124** | 0.014 | 0.014 | - | - | 0.017 | 0.017 | - | - |
|  | **129** | - | - | 0.009 | 0.009 | - | - | - | - |
|  | **NULL** | NA | 0.154 | NA | 0.013 | NA | 0.288 | NA | 0.165 |
| **Ham107** | **262** | 0.132 | 0.132 | - | - | - | - | - | - |
|  | **264** | 0.500 | 0.451 | - | - | - | - | - | - |
|  | **265** | 0.013 | 0.013 | 0.008 | 0.008 | 0.013 | 0.013 | 0.209 | 0.184 |
|  | **266** | 0.105 | 0.077 | - | - | 0.013 | 0.013 | 0.372 | 0.340 |
|  | **268** | 0.066 | 0.066 | - | - | 0.075 | 0.056 | - | - |
|  | **269** | 0.053 | 0.042 | 0.017 | 0.017 | 0.050 | 0.050 | - | - |
|  | **270** | 0.026 | 0.014 | - | - | 0.025 | 0.013 | 0.023 | 0.012 |
|  | **274** | 0.013 | 0.013 | 0.008 | 0.008 | - | - | - | - |
|  | **276** | 0.026 | 0.026 | 0.195 | 0.190 | 0.212 | 0.194 | - | - |
|  | **278** | - | - | 0.042 | 0.038 | - | - | 0.012 | 0.012 |
|  | **280** | 0.026 | 0.026 | 0.424 | 0.418 | 0.525 | 0.478 | 0.047 | 0.047 |
|  | **282** | - | - | 0.263 | 0.260 | 0.050 | 0.050 | 0.151 | 0.137 |
|  | **284** | 0.039 | 0.039 | 0.025 | 0.025 | 0.037 | 0.038 | 0.186 | 0.159 |
|  | **286** | - | - | 0.008 | 0.008 | - | - | - | - |
|  | **289** | - | - | 0.008 | 0.008 | - | - | - | - |
|  | **NULL** | NA | 0.100 | NA | 0.018 | NA | 0.097 | NA | 0.109 |
| **ham116** | **273** | 0.551 | 0.459 | - | - | - | - | - | - |
|  | **275** | 0.051 | 0.040 | 0.030 | 0.020 | 0.057 | 0.044 | 0.274 | 0.273 |
|  | **277** | 0.013 | 0.013 | - | - | - | - | 0.107 | 0.107 |
|  | **282** | 0.026 | 0.026 | - | - | - | - | - | - |
|  | **284** | - | - | - | - | 0.100 | 0.075 | 0.131 | 0.131 |
|  | **286** | 0.051 | 0.040 | 0.640 | 0.509 | 0.600 | 0.437 | 0.155 | 0.154 |
|  | **288** | 0.128 | 0.099 | 0.100 | 0.083 | 0.014 | 0.014 | 0.024 | 0.024 |
|  | **290** | 0.115 | 0.106 | 0.190 | 0.127 | 0.057 | 0.057 | 0.274 | 0.273 |
|  | **292** | 0.026 | 0.026 | 0.020 | 0.010 | 0.171 | 0.113 | - | - |
|  | **294** | - | - | 0.020 | 0.010 | - | - | - | - |
|  | **296** | 0.026 | 0.026 | - | - | - | - | - | - |
|  | **298** | 0.013 | 0.013 | - | - | - | - | 0.036 | 0.036 |
|  | **NULL** | NA | 0.154 | NA | 0.241 | NA | 0.260 | NA | 0.001 |
| **ham120** | **183** | 0.050 | 0.030 | - | - | - | - | - | - |
|  | **193** | 0.537 | 0.507 | 0.018 | 0.018 | 0.013 | 0.013 | 0.326 | 0.326 |
|  | **195** | - | - | 0.062 | 0.056 | - | - | - | - |
|  | **197** | 0.013 | 0.013 | 0.009 | 0.009 | - | - | - | - |
|  | **199** | 0.013 | 0.013 | - | - | - | - | 0.012 | 0.012 |
|  | **203** | 0.050 | 0.050 | - | - | 0.062 | 0.063 | 0.023 | 0.023 |
|  | **205** | 0.013 | 0.013 | 0.009 | 0.009 | - | - | - | - |
|  | **207** | - | - | 0.018 | 0.018 | 0.050 | 0.050 | - | - |
|  | **209** | 0.013 | 0.013 | 0.009 | 0.009 | - | - | - | - |
|  | **211** | 0.025 | 0.025 | 0.750 | 0.699 | 0.550 | 0.550 | 0.337 | 0.337 |
|  | **213** | 0.013 | 0.013 | 0.045 | 0.045 | 0.163 | 0.163 | - | - |
|  | **215** | 0.050 | 0.050 | 0.062 | 0.049 | 0.087 | 0.088 | 0.163 | 0.163 |
|  | **217** | 0.075 | 0.067 | 0.018 | 0.018 | 0.050 | 0.050 | 0.140 | 0.140 |
|  | **219** | 0.062 | 0.063 | - | - | 0.013 | 0.013 | - | - |
|  | **221** | 0.087 | 0.088 | - | - | 0.013 | 0.013 | - | - |
|  | **NULL** | NA | 0.058 | NA | 0.071 | NA | - | NA | - |
| **ham123** | **149** | - | - | 0.035 | 0.035 | 0.039 | 0.039 | - | - |
|  | **151** | - | - | 0.035 | 0.035 | - | - | 0.205 | 0.184 |
|  | **155** | - | - | 0.018 | 0.018 | - | - | - | - |
|  | **157** | 0.103 | 0.103 | 0.018 | 0.018 | 0.079 | 0.062 | - | - |
|  | **159** | 0.224 | 0.180 | 0.377 | 0.368 | 0.316 | 0.299 | 0.013 | 0.013 |
|  | **161** | 0.276 | 0.236 | 0.175 | 0.167 | 0.066 | 0.057 | 0.218 | 0.198 |
|  | **163** | 0.172 | 0.136 | 0.193 | 0.189 | 0.276 | 0.273 | 0.513 | 0.446 |
|  | **165** | 0.103 | 0.076 | 0.070 | 0.066 | 0.105 | 0.098 | - | - |
|  | **166** | - | - | 0.009 | 0.009 | - | - | - | - |
|  | **167** | 0.121 | 0.108 | 0.044 | 0.044 | 0.026 | 0.026 | - | - |
|  | **169** | - | - | 0.018 | 0.018 | 0.013 | 0.013 | 0.051 | 0.051 |
|  | **171** | - | - | 0.009 | 0.009 | 0.053 | 0.053 | - | - |
|  | **173** | - | - | - | - | 0.026 | 0.026 | - | - |
|  | **NULL** | NA | 0.161 | NA | 0.025 | NA | 0.053 | NA | 0.107 |
| **ham141** | **207** | - | - | 0.019 | 0.019 | 0.031 | 0.017 | - | - |
|  | **209** | 0.031 | 0.031 | 0.287 | 0.287 | 0.016 | 0.016 | 0.125 | 0.125 |
|  | **211** | 0.062 | 0.048 | 0.185 | 0.185 | 0.250 | 0.229 | 0.312 | 0.312 |
|  | **213** | 0.047 | 0.032 | 0.213 | 0.213 | 0.172 | 0.155 | 0.113 | 0.113 |
|  | **215** | 0.016 | 0.016 | 0.037 | 0.037 | 0.344 | 0.314 | 0.037 | 0.038 |
|  | **217** | 0.031 | 0.031 | 0.204 | 0.204 | 0.062 | 0.050 | 0.013 | 0.013 |
|  | **219** | 0.031 | 0.016 | 0.028 | 0.028 | 0.078 | 0.078 | - | - |
|  | **221** | - | - | 0.009 | 0.009 | 0.047 | 0.047 | 0.150 | 0.150 |
|  | **222** | 0.156 | 0.106 | 0.009 | 0.009 | - | - | - | - |
|  | **223** | 0.031 | 0.016 | - | - | - | - | - | - |
|  | **224** | 0.297 | 0.235 | - | - | - | - | 0.075 | 0.075 |
|  | **225** | - | - | 0.009 | 0.009 | - | - | - | - |
|  | **226** | 0.094 | 0.080 | - | - | - | - | - | - |
|  | **228** | 0.062 | 0.048 | - | - | - | - | - | - |
|  | **230** | 0.094 | 0.067 | - | - | - | - | - | - |
|  | **234** | 0.016 | 0.016 | - | - | - | - | 0.025 | 0.025 |
|  | **236** | 0.016 | 0.016 | - | - | - | - | 0.150 | 0.150 |
|  | **242** | 0.016 | 0.016 | - | - | - | - | - | - |
|  | **NULL** | NA | 0.226 | NA | - | NA | 0.094 | NA | - |
| **ham146** | **128** | 0.064 | 0.064 | - | - | 0.141 | 0.134 | 0.012 | 0.012 |
|  | **130** | 0.115 | 0.115 | - | - | - | - | - | - |
|  | **132** | 0.026 | 0.026 | 0.444 | 0.438 | - | - | 0.314 | 0.310 |
|  | **134** | 0.628 | 0.627 | 0.481 | 0.475 | 0.718 | 0.701 | 0.570 | 0.564 |
|  | **136** | 0.077 | 0.077 | - | - | 0.038 | 0.038 | 0.012 | 0.012 |
|  | **138** | - | - | 0.028 | 0.028 | - | - | - | - |
|  | **139** | 0.013 | 0.013 | - | - | - | - | - | - |
|  | **140** | - | - | - | - | 0.013 | 0.013 | 0.023 | 0.023 |
|  | **142** | 0.013 | 0.013 | 0.009 | 0.009 | - | - | - | - |
|  | **145** | 0.038 | 0.038 | 0.009 | 0.009 | - | - | - | - |
|  | **147** | 0.026 | 0.026 | - | - | - | - | - | - |
|  | **149** | - | - | 0.009 | 0.009 | - | - | - | - |
|  | **151** | - | - | 0.009 | 0.009 | 0.077 | 0.077 | 0.070 | 0.066 |
|  | **153** | - | - | 0.009 | 0.009 | 0.013 | 0.013 | - | - |
|  | **NULL** | NA | 0.001 | NA | 0.013 | NA | 0.024 | NA | 0.013 |
| **ham150** | **154** | 0.062 | 0.063 | - | - | 0.079 | 0.079 | 0.286 | 0.286 |
|  | **158** | - | - | 0.151 | 0.147 | 0.039 | 0.028 | 0.119 | 0.119 |
|  | **160** | 0.025 | 0.025 | 0.009 | 0.009 | - | - | - | - |
|  | **162** | 0.200 | 0.200 | 0.415 | 0.390 | 0.053 | 0.053 | 0.119 | 0.119 |
|  | **164** | 0.338 | 0.337 | 0.349 | 0.322 | 0.500 | 0.454 | 0.107 | 0.107 |
|  | **166** | 0.325 | 0.325 | - | - | 0.211 | 0.190 | 0.119 | 0.119 |
|  | **168** | 0.025 | 0.025 | 0.066 | 0.066 | - | - | 0.202 | 0.202 |
|  | **170** | 0.025 | 0.025 | 0.009 | 0.009 | 0.118 | 0.091 | 0.048 | 0.048 |
|  | **NULL** | NA | - | NA | 0.057 | NA | 0.106 | NA | - |
| **ham181** | **194** | - | - | 0.009 | 0.009 | 0.013 | 0.013 | - | - |
|  | **196** | 0.025 | 0.025 | 0.296 | 0.296 | 0.100 | 0.100 | 0.107 | 0.107 |
|  | **198** | 0.025 | 0.025 | 0.009 | 0.009 | - | - | - | - |
|  | **200** | 0.013 | 0.013 | 0.028 | 0.028 | 0.037 | 0.027 | 0.155 | 0.155 |
|  | **208** | - | - | - | - | 0.025 | 0.025 | - | - |
|  | **210** | - | - | 0.028 | 0.028 | 0.087 | 0.080 | - | - |
|  | **212** | 0.050 | 0.050 | 0.019 | 0.019 | 0.013 | 0.013 | 0.083 | 0.083 |
|  | **214** | 0.212 | 0.203 | 0.259 | 0.259 | 0.263 | 0.240 | 0.036 | 0.036 |
|  | **216** | 0.212 | 0.194 | 0.139 | 0.139 | 0.388 | 0.360 | - | - |
|  | **218** | 0.087 | 0.088 | 0.176 | 0.176 | 0.013 | 0.013 | 0.226 | 0.226 |
|  | **219** | - | - | - | - | 0.013 | 0.013 | - | - |
|  | **220** | 0.100 | 0.093 | - | - | 0.037 | 0.038 | 0.143 | 0.143 |
|  | **222** | 0.100 | 0.093 | - | - | 0.013 | 0.013 | 0.238 | 0.238 |
|  | **224** | 0.125 | 0.119 | 0.028 | 0.028 | - | - | 0.012 | 0.012 |
|  | **226** | 0.037 | 0.027 | 0.009 | 0.009 | - | - | - | - |
|  | **228** | 0.013 | 0.013 | - | - | - | - | - | - |
|  | **NULL** | NA | 0.058 | NA | 0.000 | NA | 0.069 | NA | - |
| **ham184** | **169** | 0.043 | 0.043 | 0.027 | 0.027 | - | - | 0.139 | 0.136 |
|  | **171** | - | - | 0.073 | 0.073 | - | - | - | - |
|  | **173** | 0.057 | 0.057 | 0.018 | 0.018 | - | - | 0.069 | 0.069 |
|  | **175** | 0.014 | 0.014 | 0.200 | 0.200 | 0.292 | 0.258 | 0.056 | 0.056 |
|  | **177** | 0.057 | 0.052 | 0.273 | 0.273 | 0.361 | 0.332 | 0.458 | 0.456 |
|  | **179** | 0.271 | 0.267 | 0.173 | 0.173 | - | - | 0.014 | 0.014 |
|  | **180** | 0.086 | 0.086 | 0.155 | 0.155 | 0.028 | 0.015 | 0.236 | 0.234 |
|  | **182** | 0.100 | 0.100 | 0.036 | 0.036 | 0.167 | 0.140 | - | - |
|  | **184** | 0.057 | 0.052 | 0.009 | 0.009 | 0.125 | 0.106 | 0.028 | 0.028 |
|  | **186** | 0.029 | 0.029 | 0.027 | 0.027 | 0.028 | 0.028 | - | - |
|  | **188** | 0.086 | 0.086 | - | - | - | - | - | - |
|  | **190** | 0.071 | 0.071 | 0.009 | 0.009 | - | - | - | - |
|  | **192** | 0.029 | 0.029 | - | - | - | - | - | - |
|  | **194** | 0.014 | 0.014 | - | - | - | - | - | - |
|  | **196** | 0.071 | 0.071 | - | - | - | - | - | - |
|  | **204** | 0.014 | 0.014 | - | - | - | - | - | 0.007 |
|  | **NULL** | NA | 0.014 | NA | - | NA | 0.122 | NA | - |
| **lchu06** | **170** | 0.175 | 0.153 | - | - | - | - | - | - |
|  | **171** | 0.037 | 0.026 | 0.384 | 0.379 | 0.061 | 0.061 | - | - |
|  | **174** | - | - | 0.009 | 0.009 | 0.024 | 0.024 | - | - |
|  | **175** | 0.013 | 0.013 | 0.348 | 0.344 | 0.512 | 0.441 | 0.107 | 0.097 |
|  | **176** | 0.100 | 0.082 | - | - | - | - | 0.107 | 0.107 |
|  | **177** | 0.013 | 0.013 | 0.009 | 0.009 | 0.110 | 0.101 | - | - |
|  | **178** | 0.037 | 0.026 | 0.018 | 0.018 | 0.122 | 0.095 | 0.190 | 0.180 |
|  | **180** | 0.113 | 0.095 | - | - | 0.073 | 0.063 | 0.226 | 0.217 |
|  | **182** | 0.013 | 0.013 | 0.027 | 0.027 | 0.012 | 0.012 | 0.024 | 0.024 |
|  | **183** | - | - | 0.009 | 0.009 | - | - | - | - |
|  | **184** | 0.025 | 0.025 | - | - | 0.037 | 0.025 | 0.310 | 0.303 |
|  | **185** | 0.100 | 0.091 | 0.009 | 0.009 | - | - | - | - |
|  | **186** | 0.100 | 0.082 | 0.116 | 0.114 | 0.024 | 0.013 | - | - |
|  | **187** | 0.075 | 0.075 | - | - | - | - | - | - |
|  | **188** | 0.050 | 0.050 | 0.071 | 0.069 | - | - | 0.036 | 0.036 |
|  | **190** | - | - | - | - | 0.024 | 0.024 | - | - |
|  | **191** | 0.075 | 0.075 | - | - | - | - | - | - |
|  | **193** | 0.025 | 0.025 | - | - | - | - | - | - |
|  | **195** | 0.050 | 0.050 | - | - | - | - | - | - |
|  | **NULL** | NA | 0.106 | NA | 0.014 | NA | 0.140 | NA | 0.036 |
|  | **-** | - | - |  | - | - | - | - | - |
|  | | | | | | | | | |
