# Supplementary material for: Natural and Anthropogenic Hybridization in Two Species of Eastern Brazilian Marmosets (Callithrix jacchus and C. penicillata)
Source: PLoS One. 2015 Jun 10;10(6):e0127268. doi: 10.1371/journal.pone.0127268 (PMC4464756; doi:10.1371/journal.pone.0127268)
Supplement: S8 Table — (DOCX) [file pone.0127268.s010.docx]

S8 Table. STRUCTURE results for 10 different simulated data sets*.* “*Q* F1” is average *q*-coefficient of F1 simulated hybrids across replicate runs of each dataset, “F1 *q-*range” is average *q*-coefficient range of F1 simulated hybrids across replicate dataset runs, “F1 CI interval” is the average 90% confidence interval of F1 simulated hybrids across replicate runs of each dataset. F2 represents simulated F2 hybrids, *C. jacchus* BC represents simulated hybrid offspring of a F1x pure *C. jacchus* backcross, and *C. penicillata* BC represents simulated hybrid offspring of a F1 x pure *C. penicillata* backcross. Columns for all other simulated hybrid classes follow same conventions as described for simulated F1 hybrids.

| **Set** | ***q* F1** | **F1 *q*-range** | **F1 CI interval** | ***q* F2** | **F2 *q* -range** | **F2 CI interval** | ***q* *C. jacchus* BC** | ***C. jacchus* BC *q-r*ange** | ***C. jacchus* BC CI interval** | ***q* *C. penicillata* BC** | ***C. penicillata* BC *q*-range** | ***C. penicillata* BC CI interval** |
| --- | --- | --- | --- | --- | --- | --- | --- | --- | --- | --- | --- | --- |
| 1 | 0.55 | 0.50-0.62 | 0.43-0.67 | 0.56 | 0.44-0.61 | 0.44-0.68 | 0.35 | 0.29-0.41 | 0.24-0.47 | 0.76 | 0.67-0.82 | 0.65- 0.86 |
| 2 | 0.55 | 0.50-0.61 | 0.42-0.66 | 0.56 | 0.46-0.61 | 0.43-0.66 | 0.35 | 0.29-0.40 | 0.27-0.49 | 0.76 | 0.67-0.82 | 0.62- 0.84 |
| 3 | 0.55 | 0.51-0.60 | 0.44-0.67 | 0.56 | 0.50-0.61 | 0.44-0.68 | 0.34 | 0.25-0.46 | 0.23-0.46 | 0.78 | 0.71-0.86 | 0.67- 0.88 |
| 4 | 0.56 | 0.46-0.64 | 0.44-0.67 | 0.55 | 0.50-0.64 | 0.43-0.67 | 0.34 | 0.25-0.38 | 0.23-0.45 | 0.73 | 0.65-0.80 | 0.62- 0.84 |
| 5 | 0.54 | 0.46-0.60 | 0.44-0.66 | 0.54 | 0.43-0.67 | 0.42-0.66 | 0.35 | 0.25-0.38 | 0.24-0.46 | 0.76 | 0.65-0.80 | 0.65- 0.86 |
| 6 | 0.55 | 0.47-0.61 | 0.43-0.67 | 0.57 | 0.52-0.64 | 0.45-0.68 | 0.37 | 0.28-0.47 | 0.26-0.49 | 0.76 | 0.68-0.81 | 0.64- 0.86 |
| 7 | 0.56 | 0.50-0.66 | 0.43-0.58 | 0.55 | 0.47-0.58 | 0.43-0.68 | 0.37 | 0.25-0.45 | 0.26-0.49 | 0.79 | 0.73-0.84 | 0.68- 0.89 |
| 8 | 0.55 | 0.48-0.57 | 0.43-0.67 | 0.57 | 0.48-0.67 | 0.45-0.69 | 0.36 | 0.27-0.42 | 0.24-0.47 | 0.76 | 0.64-0.80 | 0.65- 0.86 |
| 9 | 0.56 | 0.47-0.60 | 0.44-0.68 | 0.57 | 0.42-0.76 | 0.45-0.69 | 0.34 | 0.29-0.38 | 0.23-0.45 | 0.78 | 0.73-0.81 | 0.67- 0.88 |
| 10 | 0.56 | 0.50-0.57 | 0.44-0.68 | 0.56 | 0.45-0.68 | 0.44-0.67 | 0.35 | 0.20-0.39 | 0.24-0.46 | 0.77 | 0.66-0.82 | 0.66- 0.87 |
