# Supplementary material for: Natural and Anthropogenic Hybridization in Two Species of Eastern Brazilian Marmosets (Callithrix jacchus and C. penicillata)
Source: PLoS One. 2015 Jun 10;10(6):e0127268. doi: 10.1371/journal.pone.0127268 (PMC4464756; doi:10.1371/journal.pone.0127268)
Supplement: S9 Table — (DOCX) [file pone.0127268.s011.docx]

S9 Table. BAPS results for 5 different simulated data sets. “F1 *q*” are average *q*-coefficients of F1 simulated hybrids across replicate runs of each dataset and “F1 *q*-range” are average *q*-coefficient range of F1 simulated hybrids across replicate runs of each data set. F2 represents simulated F2 hybrids, *C. jacchus* BC represents simulated hybrid offspring of a F1x pure *C. jacchus* backcross, and *C. penicillata* BC represents simulated hybrid offspring of a F1 x pure *C. penicillata* backcross. Columns for each simulated hybrid class follow same conventions as described for simulated F1 hybrids.

| **Set** | ***q* F1** | **F1 *q*-range** | ***q* F2** | **F2 *q-* range** | ***q* *C. jacchus* BC** | ***C. jacchus* BC *q-* range** | ***q* *C. penicillata* BC** | ***C. penicillata* BC *q*- range** |
| --- | --- | --- | --- | --- | --- | --- | --- | --- |
| 1 | 0.51 | 0.45-0.56 | 0.48 | 0.40-0.53 | 0.24 | 0.18-0.28 | 0.74 | 0.62-1.00 |
| 2 | 0.49 | 0.46-0.56 | 0.45 | 0.33-0.52 | 0.26 | 0.21-0.28 | 0.76 | 0.65-0.84 |
| 3 | 0.49 | 0.43-0.60 | 0.52 | 0.45-0.61 | 0.24 | 0.15-0.28 | 0.74 | 0.68-0.83 |
| 4 | 0.51 | 0.45-0.55 | 0.52 | 0.35-0.68 | 0.24 | 0.12-0.29 | 0.77 | 0.70-0.81 |
| 5 | 0.49 | 0.43-0.55 | 0.5 | 0.42-057 | 0.24 | 0.20-0.27 | 0.75 | 0.62-1.00 |
